# Restrictive versus Liberal blood transfusion strategies for patients undergoing orthopedic surgery: a meta-analysis of randomised trials with trial sequential analysis

**DOI:** 10.1186/s13018-025-05883-0

**Published:** 2025-05-24

**Authors:** Zhou Zhou, Zefeng Xiao, Yan Luo, Tuanbiao Nie, Xuelian Xiao

**Affiliations:** 1 Department of Neurosurgery, The Affiliated Cancer Hospital of Xiangya School of Medicine, Hunan Cancer Hospital, Central South University, No. 283 Tongzipo Road, Yuelu District, Changsha, Hunan 410013 China; 2 Department of Nursing, The Affiliated Cancer Hospital of Xiangya School of Medicine, Hunan Cancer Hospital, Central South University, No. 283 Tongzipo Road, Yuelu District, Changsha, Hunan 410013 China; 3 Department of Orthopedics, Xiangya Hospital, Central South University, No. 87, Xiangya Road, Changsha, Hunan China; 4 Clinical Medicine Eight-Year Program, Xiangya Hospital, Central South University, Central South University, No. 87, Xiangya Road, Changsha, Hunan China; 5 Department of Medical Administration, The Affiliated Cancer Hospital of Xiangya School of Medicine, Hunan Cancer Hospital, Central South University, No. 283 Tongzipo Road, Yuelu District, Changsha, Hunan 410013 China

**Keywords:** Blood transfusion, Orthopedic patients, Meta-analysis, Trial sequential analysis, Liberal transfusion, Restrictive transfusion

## Abstract

**Background:**

A meta-analysis was conducted to explore the prognostic differences of restrictive blood transfusion (RBT) versus liberal blood transfusion (LBT) strategies in orthopedic patients.

**Methods:**

A comprehensive search was performed in PubMed, Embase, Cochrane Central Register of Controlled Trials, Embase, and clinicaltrials.gov up to 20 October 2024. The quality of included studies was assessed according to Cochrane risk of bias, and quality of evidence was assessed using the GRADE system. We performed sensitivity and publication bias analyses and used trial sequential analysis (TSA) to assess the risk of random error in the analysis results.

**Results:**

19 studies involving 7833 patients were included in the analysis. Compared with LBT, RBT reduced transfusion rate and increased the occurrence of cardiovascular events (RR = 1.44; 95% CI: 1.15–1.80, *P* = 0.001; *I*^*2*^ = 0%), mainly increased myocardial infarction (RR = 1.70; 95% CI: 1.16–2.48, *P* = 0.006; *I*^*2*^ = 0%) rather than congestive heart failure. There were no significant differences between transfusion strategies in infection, thrombotic events, mortality, delirium and length of hospitalization. Results of subgroup analyses indicate that in patients at high risk for cardiovascular disease, RBT increases the risk of myocardial infarction and length of hospitalization. In addition, RBT are associated with lower overall infection rates and shorter length of hospitalization after joint replacement or revision surgery; and are associated with an increased risk of myocardial infarction after fracture repair surgery (RR = 1.79; 95% CI: 1.21–2.65, *P* = 0.004). The TSA results show that transfusion rate and mortality (≥ 60 days) have reached the required information size. However, the evidence regarding the efficacy for the remaining outcomes analyzed remains inconclusive, likely due to insufficient numbers of patients in the existing studies.

**Conclusions:**

Compared with LBT, RBT increases the risk of cardiovascular events in orthopedic patients but does not affect adverse outcomes such as infection, thrombotic events, mortality, and delirium.

**Trial registration:**

No patients were involved in this study.

**Graphical Abstract:**

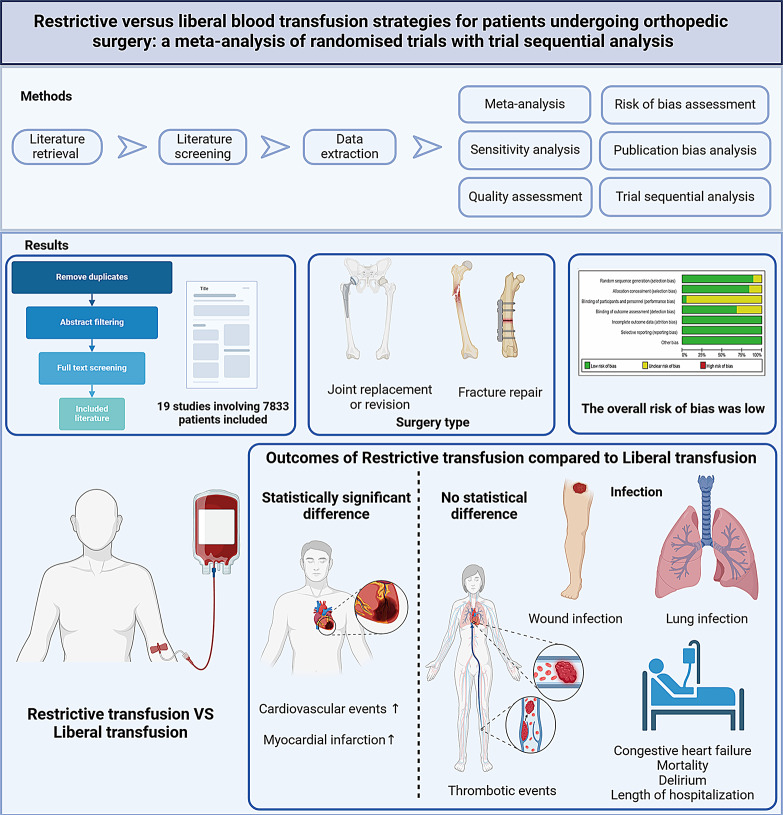

**Supplementary Information:**

The online version contains supplementary material available at 10.1186/s13018-025-05883-0.

## Background

Red blood cell (RBC) transfusion is a common treatment used to rapidly enhance the oxygen-carrying capacity of the blood, with approximately 118 million units collected worldwide each year [1, 2]. Delayed blood transfusion can lead to ischemic tissue injuries in patients, thereby increasing mortality. Conversely, transfusion may also elevate the incidence of various adverse events and associated hospitalization costs [3]. Consequently, several published guidelines have discussed the appropriate threshold for RBC transfusion, generally recommending a restrictive threshold of 70–80 g/L [1, 4–8]. While RBT practices are primarily based on hemoglobin levels in most orthopedic surgeries, current blood transfusion strategies exhibit significant variability. The optimal approach to blood transfusion remains a subject of controversy, as there are no universally applicable standards, and transfusion-related adverse events still cannot be overlooked [9, 10]. Multiple systematic reviews and meta-analyses have evaluated the effectiveness of blood transfusion (RBT) (initiated mainly when hemoglobin level reach 8.0 g/dL or in cases of symptomatic anemia) versus liberal blood transfusion (LBT) (initiated mainly when hemoglobin level reach 10.0 g/dL) in orthopedic surgery patients [11–16]. However, results have been inconsistent and inconclusive across different patient features and clinical surgery types [11–16].

In recent years, several pertinent randomized controlled trials (RCTs) have been published [17–19], including a study by Quaranta et al. [20] showing that orthogeriatric multidisciplinary care in elderly hip fracture patients improved postoperative hemoglobin and reduced transfusions, especially after hemiarthroplasty and highlighting the need for comprehensive reviews and meta-analyses to elucidate the clinical outcomes associated with different transfusion strategies. This study aims to thoroughly evaluate the safety and prognostic differences between RBT and LBT practices in orthopedic patients. We conducted meta-analyses and subgroup analyses to identify patient characteristics and surgical approaches that may influence these prognostic differences. Notably, we employed trial sequential analysis (TSA) to assess both the adequacy and reliability of the accumulated published evidence, as well as to evaluate the heterogeneity of study results and the potential for publication bias.

## Methods

### Registration and protocol

This meta-analysis was based on the Cochrane Handbook, Preferred Reporting Items for Systematic Reviews and Meta-Analyses (PRISMA) guidelines [21] and assessment of multiple systematic reviews (AMSTAR) guidelines [22]. The protocol of this study has been prospectively registered and published in the PROSPERO, and there has been no deviation from the protocol.

### Search strategy

PubMed, Web of Science, EMBASE, Cochrane Central Register of Controlled Trials and ClinicalTrials.gov (http://www.controlledtrials.com/) were searched up to 20 October 2024. We constructed search terms using appropriate combinations of the following combined search terms with MeSH terms and adjusted accordingly for different databases: (blood transfusion) AND (orthopedic surgery) AND (randomized controlled trials). Details of the search strategy are provided in the Supplementary materials.

### Criteria for inclusion and exclusion

Inclusion criteria: [1] Randomized controlled trials; [2] Study participants were patients undergoing orthopedic surgery; [3] Study groups included at least a RBT group and a LBT group; [4] Studies reported at least one primary or secondary outcome of this meta-analysis.

Exclusion criteria: [1] Conference abstracts, cohort studies, meta-analyses, editorials, and reviews; [2] Duplicate publications; [3] No detailed extractable data reported; [5] Animal experiments.

### Literature screen and data collection

After searching the literature, two authors independently screened the literature, first excluded duplicate literature, then read the abstracts to exclude irrelevant studies and finally reviewed the full text to determine the final included articles. Two authors then independently extracted data, including article characteristics and outcome data, from studies that met the inclusion criteria. When disagreements occurred, a third author joined the discussion to reach consensus.

### Outcomes

Our primary outcomes were overall infection rates and cardiovascular event rates. For the overall infection, we considered all infections reported in the study. Cardiovascular events include myocardial infarction (MI), congestive heart failure (CHF) and arrhythmia (For relevant definitions, refer to the Supplementary Table 1. Secondary outcomes included lung infection, wound infection, MI, CHF, thromboembolic events, cerebrovascular accidents, overall mortality, transfusion rates, delirium and length of hospitalization. In the subgroup analysis, the definitions of patients at high risk for cardiovascular disease and ordinary patients are shown in the Supplementary Table 2.

### Quality assessment and data synthesis and analysis

The risk of bias of included studies was assessed according to the Cochrane Collaboration tool. Following the GRADE manual, GRADEpro 3.6 was used to assess the quality of evidence for meta-analyses. Two authors independently assessed the risk of bias and quality of evidence, and in case of inconsistencies, a third author participated in a discussion to obtain the final results. Dichotomous variables and continuous variables were expressed as risk ratio (RR) and standard mean difference (SMD), respectively, with a confidence interval (CI) of 95%. *I*^*2*^ is a quantitative measure used to assess the degree of heterogeneity. *I*^*2*^ < 50% is considered to have insignificant heterogeneity, while *I*^*2*^ > 50% indicates that heterogeneity may be significant. *I*^*2*^ > 75% indicates high heterogeneity. When *I*^*2*^ < 50%, the fixed effects model is used for analysis; otherwise, the random effects model is used. Sensitivity analysis is used to evaluate the impact of each study on the final total effect. Publication bias was assessed using Egger’s test, and *p* < 0.05 was considered statistically significant. If p was ≥ 0.5 and there was sufficient data, the trim-and-fill method was used to adjust for this bias [23]. RevMan 5.4 software and Stata 14.0 software were used for analysis.

### Trial sequential analysis

We performed TSA to assess whether the currently accumulated clinical data are sufficient to determine the conclusiveness of the results while assessing the risk of random error [24]. The relative risk reduction was predefined as 20%, the α value was set at 5%, the β value was set at 20% (80% power), and the control event rate was calculated based on the restricted RBC transfusion group. Based on the above information, we calculated the required information size (RIS) for each result using TSA Viewer 0.9 beta software.

## Results

### Literature retrieval and screening

**Fig. 1:**
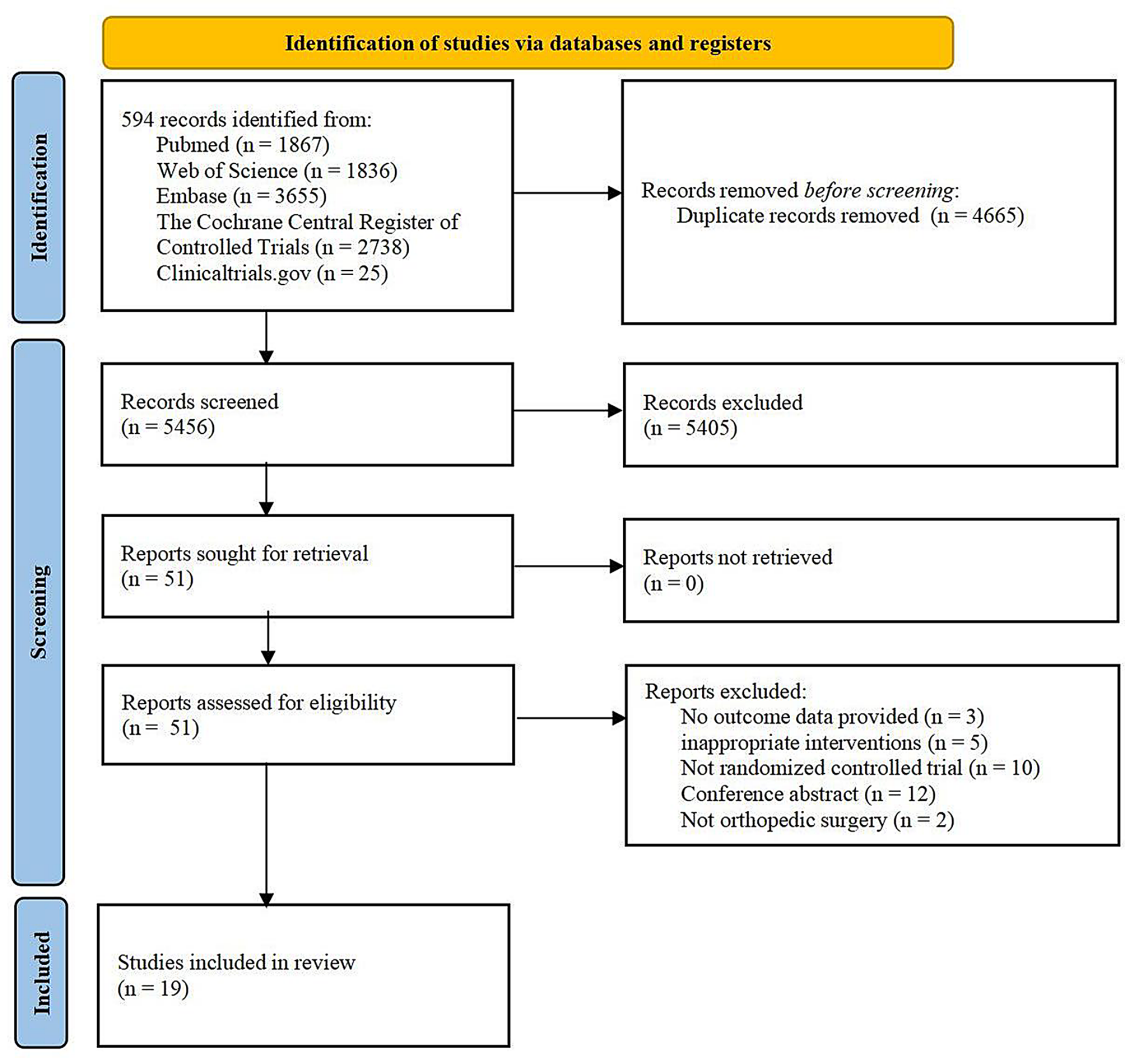
Literature screening flow chart

**Fig. 2:**
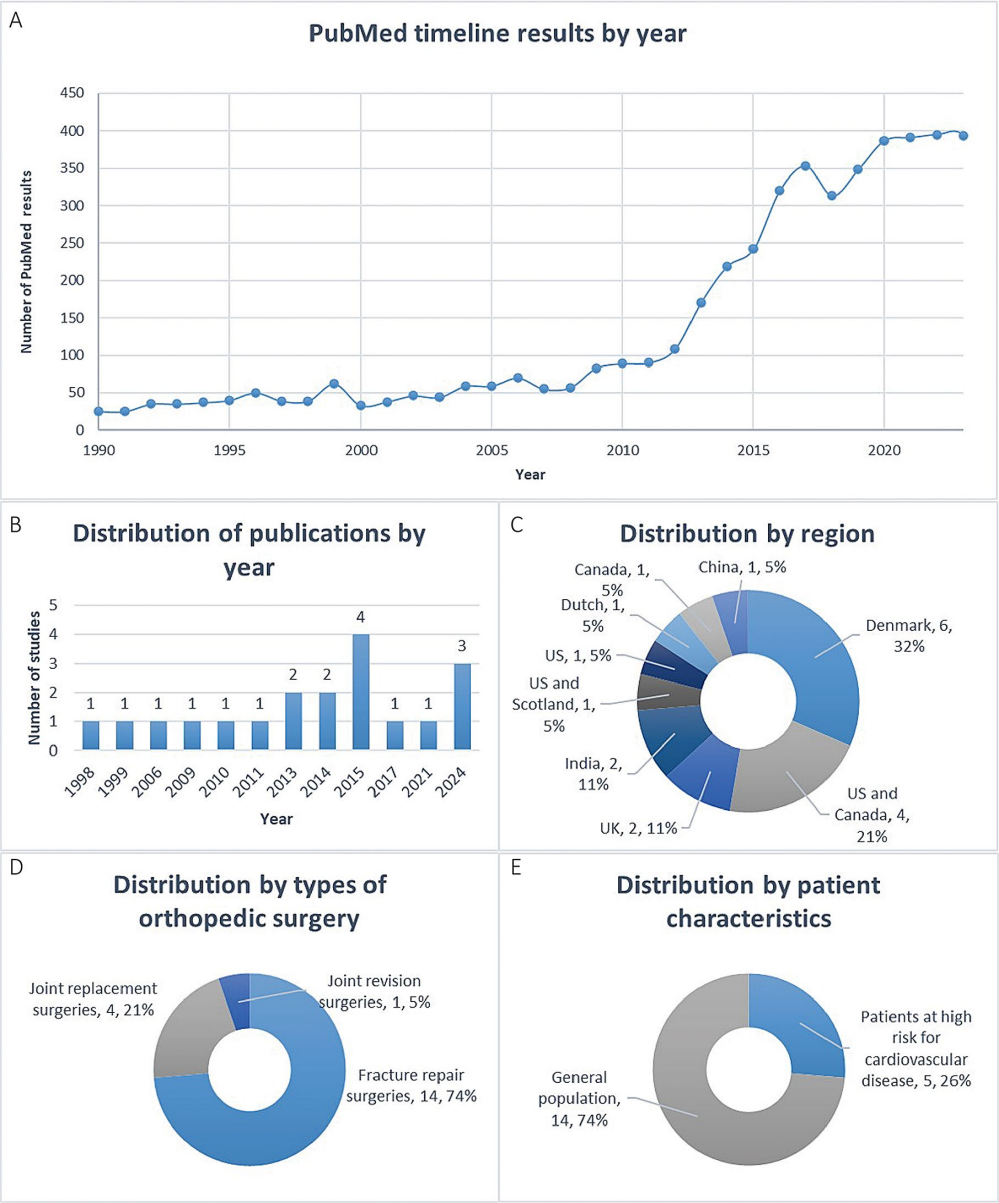
An overview of studies characteristics, including distribution of (**A**) PubMed timeline results by year (**B**) publications by year (**C**) region (**D**) types of orthopedic surgery (**E**) and patient characteristics

The literature screening flow chart is shown in Fig. 1. After the database search, 10,121 potentially relevant publications were detected. Among them, 4665 duplicate documents were deleted. 5405 articles were excluded because they did not meet the title and abstract review. 32 articles were excluded due to ineligibility after reading the full text (see the supplementary materials for reasons of exclusion), and 19 studies were finally included in this meta-analysis [17–19, 25–40]. We also noticed that the search results of PubMed showed that the number of publications related to blood transfusion and orthopedic surgery has increased rapidly in recent years (Fig. 2A), indicating that it is currently a hot topic.

### Study characteristics

Table 1 summarizes the characteristics of the included studies, and Table 2 summarizes the included participant characteristics and transfusion information. These 19 studies were conducted from 1998 to 2024 (Fig. 2B) and were carried out in the United States, Canada, Denmark, the United Kingdom, India and other places (Fig. 2C). The total number of patients was 7833, of which 3918 were in the RBT group and the rest were in the LBT group. Fourteen studies were fracture repair surgeries [17–19, 25, 28, 30–32, 35–40], four studies were joint replacement surgeries [26, 27, 29, 33], and one study was joint revision surgeries [34](Fig. 2D). Five studies included patients at high risk for cardiovascular disease [30–32, 35, 40], and the remainder were from the general population (Fig. 2E). In the included studies, hemoglobin thresholds ranged from 5.5 g/dl to 9.7 g/dl in the RBT group and from 7.0 g/dl to 10.0 g/dl in the LBT group. Of note, two study explored the relative safety of more conservative RBT (threshold 5.5 g/dL) versus more liberal LBT (threshold 7.0 g/dL) in asymptomatic orthopedic patients during the initial resuscitation period, which are extreme thresholds [18, 19]. Five studies used leukocyte-reduced blood components [27–30, 35]. The included trials all used perioperative blood transfusion, including before, during and after surgery.

**Table 1:** Characteristics of included studies

Study	Region	Participants (*n*)	Type of surgery	Restrictive blood transfusion strategies		Liberal blood transfusion strategies		Follow-up
				Number of people	Transfusion threshold	Number of people	Transfusion threshold	
Carson 1998	US and Scotland	84	Hip fracture repair	42	Hb < 8.0 g/dl or symptomatic anemia	42	Hb < 10.0 g/dl	Postoperative 60 days
Lotke 1999	US	152	Total knee replacement	62	Hb < 9.0 g/dL	65	Beginning immediately after surgery	Postoperative 4 days
Grover 2006	Southeast England	260	Total hip or knee replacement	130	Hb < 8.0 g/dl, maintenance range, 8.0–9.5 g/dl	130	Hb < 10.0 g/dl, mainte_x005f_x005f_x005f_x0002_nance range, 10.0–12.0 g/dl	Postoperative 5 days
Foss 2009	Denmark	120	Hip fracture repair	60	Hb < 8.0 g/dl	60	Hb < 10.0 g/dl	Postoperative 3 days
So-Osman 2010	Dutch	619	Total hip or knee replacement	309	Threshold range,6.4–9.7 g/dl	310	Varied by hospital, age and condition of patients, symptoms and time	14 days after surgery or at final discharge
Carson 2011	US and Canada	2016	Hip fracture repair	1009	Symptomatic anemia or if Hb < 8.0 g/dL	1007	Hb < 10.0 g/dl	Postoperative 60 days
Carson 2015	US and Canada	2016	Hip fracture repair	1009	Symptomatic anemia or if Hb < 8.0 g/dL	1007	Hb < 10.0 g/dl	Median follow-up of 3 years
Zhang 2024	US and Canada	805	Hip fracture repair	403	Symptomatic anemia or if Hb < 8.0 g/dL	402	Hb < 10.0 g/dl	Postoperative 30 and 60 days
Gruber-Baldini 2013	US and Canada	139	Hip fracture repair	72	Symptoms or ≤ 8 g/dL	66	≤ 10 g/dL	Postrandomization days 5
Parker 2013	Canada	200	Hip fracture repair	100	8.0–9.5 g/dl and symptomatic anemia	100	8.0–9.5 g/dl	Postoperative 1 year
Fan 2014	China	192	Total hip replacement	96	Symptomatic anemia or Hb < 8.0 g/dl,	96	maintenance ≥ 10 g/dl	Postoperative 7 days
Nielsen 2014	Denmark	66	Hip revision	33	Hb < 7.3 g/dL	33	Hb < 8.9 g/dL	Postoperative 30 days
Gregersen 2015a	Denmark	284	Hip fracture repair	144	Hb < 9.7 g/dl	140	Hb < 11.3 g/dl	Postoperative 90 days
Gregersen 2015b	Denmark	284	Hip fracture repair	144	Hb < 9.7 g/dl	140	Hb < 11.3 g/dl	Postoperative 10 and 30 days
Gregersen 2015c	Denmark	157	Hip fracture repair	80	Hb < 9.7 g/dl	77	Hb < 11.3 g/dl	Postoperative 30 days and 1 year
Blandfort 2017	Denmark	179	Hip fracture repair	89	Hb < 9.7 g/dl	90	Hb < 11.3 g/dl	Postoperative 10 days
Gillies 2021	UK	62	Neck of femur fracture repair	36	Hb ≤ 7.0 g/dl	26	7.0–9.0 g/dl	Postoperative 30 and 60 days
Mullis 2024a	India	99	Fracture repair	50	Hb < 5.5 g/dL	49	Hb < 7.0 g/dL	2 weeks and 30 days after injury
Mullis 2024b	India	99	Fracture repair	50	Hb < 5.5 g/dL	49	Hb < 7.0 g/dL	6 months and 1 year

Abbreviation: Hb: hemoglobin

**Table 2:** Characteristics and transfusion information of patients included in the study

Study	Age		Number of males and females (M: F)		Patient characteristics	RBCs (type/suspension/leucocyte reduced)	Baseline Hemoglobin		RBC Transfused		Transfusion rate	
	*R*	F	*R*	F			*R*	F	*R*	F	*R*	F
Carson 1998	83.3 ± 10.8	81.3 ± 8.1	9:33	11:31	Patients with primary hip fracture	Allogen/NA/NA	(9.1 ± 0.6) g/dl	(9.1 ± 0.6) g/dl	0 (Median) (IQR 0–2) units	2 (median) (IQR 1–2)	19 (45.2%)	41 (97.6%)
Lotke 1999	68.7	69.7	20:42	20:45	Total knee arthroplasty, osteoarthritis in 88% patients	Allogen/NA/NA	NA	NA	NA	NA	NA	NA
Grover 2006	70.7 ± 7.1	71.5 ± 7.6	48:61	55:45	Elective total knee or hip arthroplasty	Allogen/NA/leucocyte reduced	(13.1 ± 1.22) g/dl	(13.6 ± 1.22) g/dl	0 (Median) (Range 0–5) units	0 (Median) (Range 0–10) units	37 (34%)	46 (43%)
Foss 2009	81 ± 7.3	81 ± 6.8	14:46	14:46	Patients with primary hip fracture	Allogen/NA/leucocyte reduced	No available but graphed	No available but graphed	1 (Median) (IQR 1–2) units	2 (Median) (IQR 1–2) units	22 (37%	44 (74%)
So-Osman 2010	70.7 ± 10.2	70.3 ± 9.7	84:215	118:186	Elective orthopaedic surgery patients	Allogen/NA/leucocyte reduced	(13.7 ± 1.4) g/dl	(13.7 ± 1.4) g/dl	0.78 (Mean) ± 1.4 (SD)	0.86 (Mean) ± 1.6 (SD)	105 (35%)	93 (31%)
Carson 2011	81.5 ± 9.0	81.8 ± 8.8	239:770	250:757	Hip fracture patients at high risk for cardiovascular disease	NA/NA/leucocyte reduced	(11.3 ± 1.5) g/dl	(11.3 ± 1.5) g/dl	0 (Median) (IQR 0–1) units	2 (Median) (IQR 1–2) units	413 (41%)	970 (97%)
Carson 2015	81.5 ± 9.0	81.8 ± 8.8	239:770	250:757	Hip fracture patients at high risk for cardiovascular disease	NA/NA/leucocyte reduced	(11.3 ± 1.5) g/dl	(11.3 ± 1.5) g/dl	0 (Median) (IQR 0–1) units	2 (Median) (IQR 1–2) units	413 (41%)	970 (97%)
Zhang 2024	82.2 ± 8.2	82.4 ± 8.5	120:283	137:265	Patients with coronary artery disease	NA/NA/NA	(11.2 ± 1.5) g/dl	(11.3 ± 1.4) g/dl	0 (Median) (IQR 0–1) units	2 (Median) (IQR 1–2) units	128 (31.8%)	110 (27.4%)
Gruber-Baldini 2013	80.6 ± 10.4	82.4 ± 7.4	25:47	12:54	Cardiovascular patients undergoing surgical hip fracture repair	NA/NA/NA	(11.9 ± 1.7) g/dl	(11.9 ± 1.3) g/dl	0 (Median) units	2 (Median) units	33(45.8%)	63 (95.4%
Parker 2013	84.2	84.4	15:85	17:83	Patients with primary hip fracture	NA/NA/NA	11.8 g/dl	11.5 g/dl	No one received a blood transfusion	All patients received a blood transfusion with a mean of 1.9 units	0 (0%)	100 (100%)
Fan 2014	75 ± 6	73 ± 7	30:64	33:59	Patients undergoing elective unilateral total hip replacement	NA/NA/NA	(12.0 ± 1.1) g/dl	(11.8 ± 1.2) g/dl	NA	NA	41(43.6%)	52 (56.5%)
Nielsen 2014	68	72	16:17	20:23	Patients undergoing elective hip revision surgery	NA/NA/NA	13.4 (10.2–15.0) g/dL	13.8 (10.5–16.3) g/dL	median with (5–95% range): 0 (0–245)	median with (5–95% range): 0 (0–245)	11 (33.3%)	16 (48.4%)
Gregersen 2015a	85.7 ± 6.9	86.9 ± 9.8	36:108	34:106	Patients with hip fracture	NA/NA/NA	(10.4 ± 1.31) g/dl	(10.3 ± 1.44) g/dl	1 (IQR 1–2) units	3 (IQR 2–5) units	24 (16%)	20 (14%)
Gregersen 2015b	85.7 ± 6.9	86.9 ± 9.8	36:108	34:106	Patients with hip fracture	NA/NA/NA	(10.4 ± 1.31) g/dl	(10.3 ± 1.44) g/dl	1 (IQR 1–2) units	3 (IQR 2–5) units	24 (16%)	20 (14%)
Gregersen 2015c	85.5 ± 6.5	87.2 ± 7.3	18:62	17:60	Patients with hip fracture	NA/NA/NA	(11.3 ± 0.1) g/dl	(12.3 ± 0.2) g/dl	1.5 (IQR 1–2.5) units	3 (IQR 2–4) units	61 (76%)	77 (100%)
Blandfort 2017	86.5 ± 6.7	88.7 ± 6.3	22:67	22:68	Patients with hip fracture	NA/NA/NA	NA	NA	NA	NA	NA	NA
Gillies 2021	82 ± 11	82 ± 11	10:26	8:16	Patients with fractured neck of femur	NA/NA/NA	(12.0 ± 1.5) g/dl	(11.5 ± 1.5) g/dl	NA	NA	24 (67%)	24 (92%)
Mullis 2024a	35.5	30	31:19	33:16	Patients with musculoskeletal trauma	NA/NA/NA	NA	NA	NA	NA	50 (100%)	46 (93.8%)
Mullis 2024b	35.5	30	31:19	33:16	Patients with musculoskeletal trauma	NA/NA/NA	NA	NA	NA	NA	50 (100%)	46 (93.8%)

R: restrictive blood transfution strategies; L: liberal blood transfution strategies; RBC: red blood cell; NA: not available; SD: standard deviation; IQR: inter-quartile range

### Risk of bias in the eligible studies and quality of evidence

**Fig. 3:**
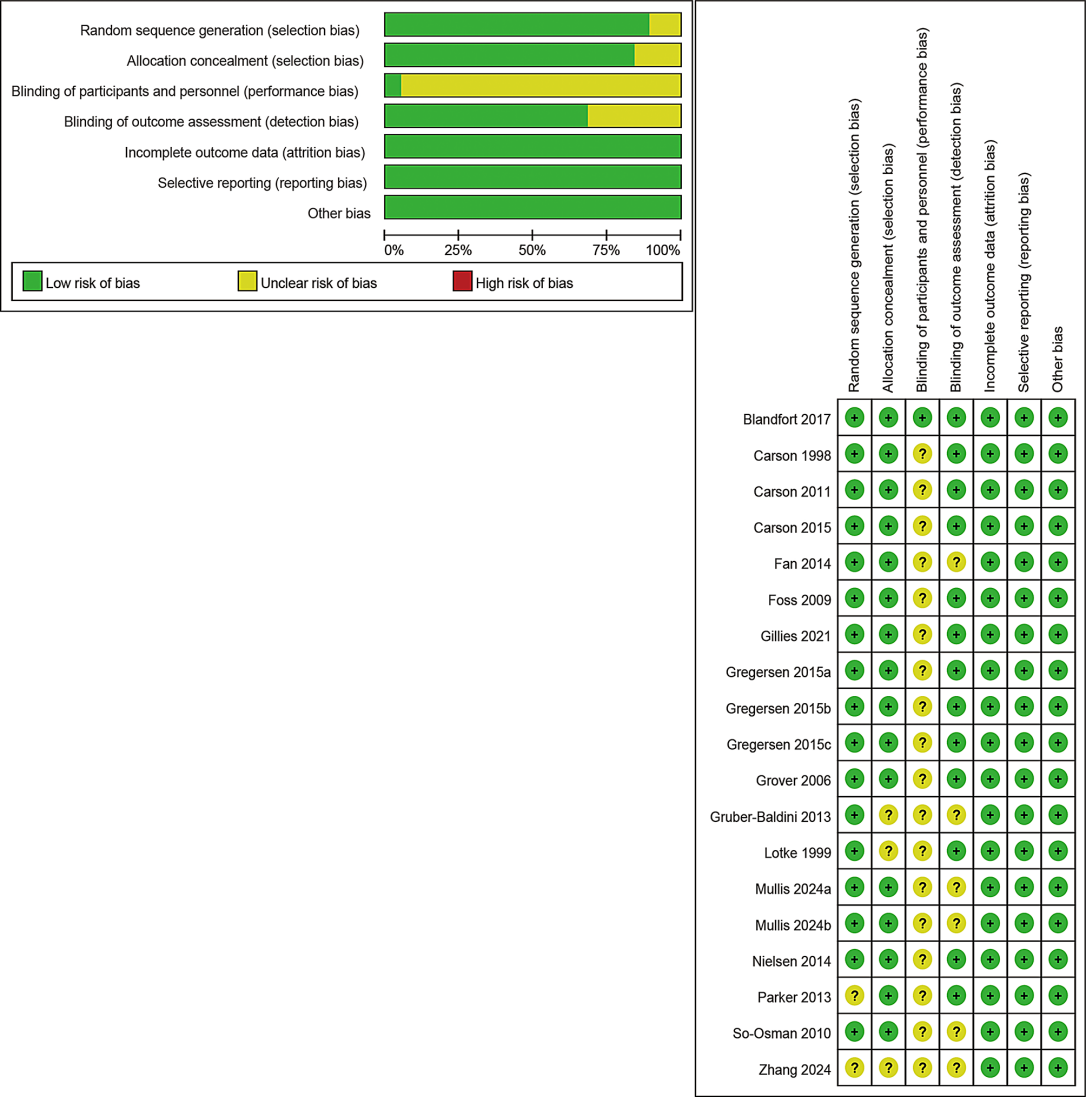
Risk of bias summary

The risk of bias of the included studies is shown in Fig. 3. 17 articles provide explicit methods for randomized sequence generation [17–19, 25–31, 33–39]. 16 studies provided actual allocation concealment procedures [17–19, 25, 27–30, 32–39]. One article reported the actual blinding procedure [39]. All articles mentioned that the authors had no conflicts of interest. Although there are some unknown risks, the overall risk of bias in the included studies was low. The quality of the evidence was assessed according to the GRADE evidence profile (see Supplementary Materials). Among the analytical results of this study, the evidence level for 3 outcomes (pulmonary infection, wound infection, CHF) was judged to be high quality, the evidence quality for blood transfusion rate was low, and the evidence quality for the remaining outcomes was moderate. Common reasons for downgrading include publication bias and high heterogeneity. Overall, the level of evidence from this meta-analysis was moderate.

### Results of meta-analysis

#### Infection

**Fig. 4:**
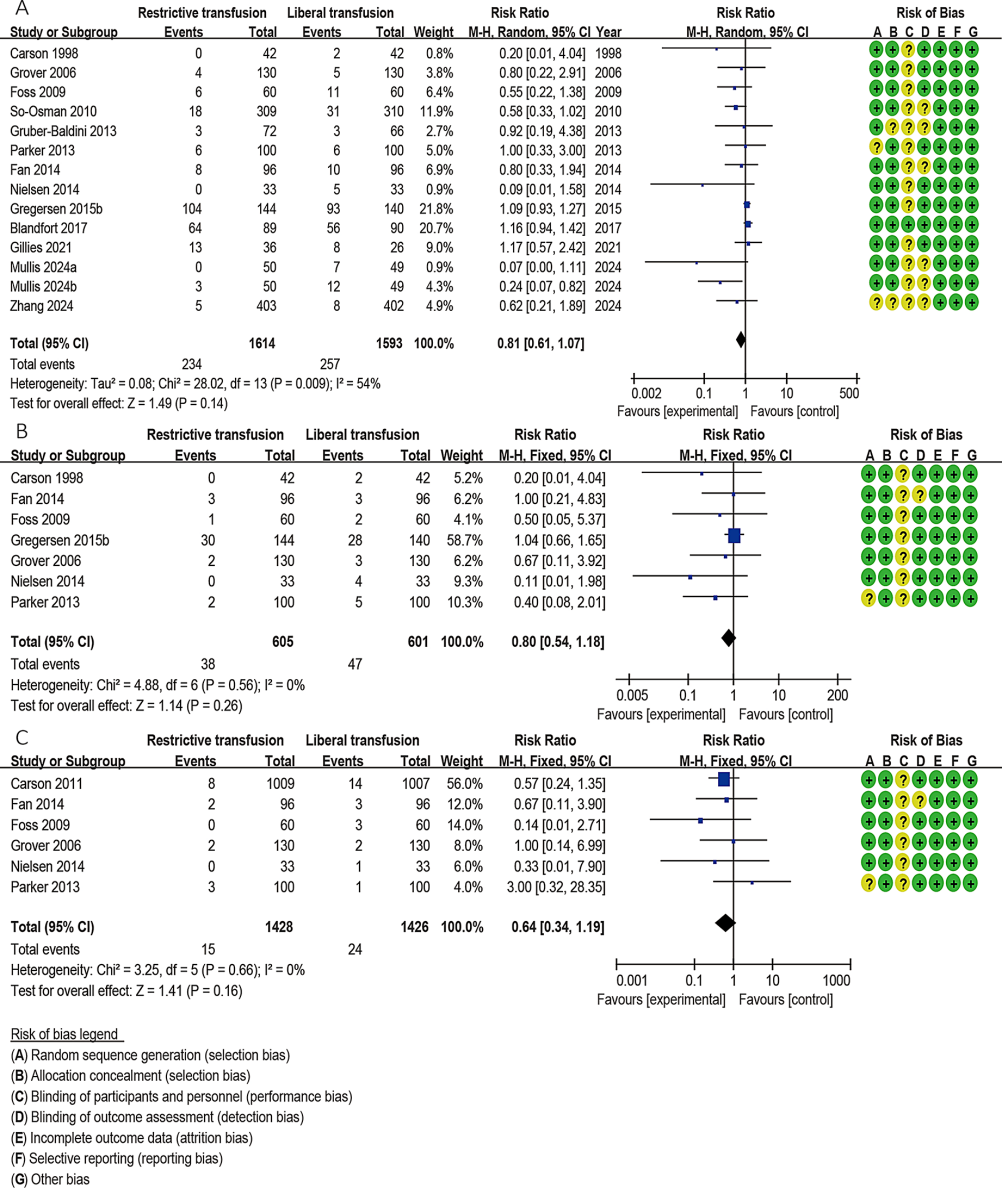
Forest plots depicting the comparison between the restrictive blood transfusion (RBT) group and liberal blood transfusion (LBT) group: (**A**) Overall infection; (**B**) Lung infection; (**C**) Wound infection

Fourteen studies including 3207 patients [17–19, 25, 27–29, 31–34, 38–40] reported overall infection. Pooled analysis with a random effects model showed that the association between transfusion strategy and infection was not statistically significant (RR = 0.81; 95% CI, 0.61–1.07, *P* = 0.14, *I*^*2*^ = 40%) (Fig. 4A). Of these studies, 7 studies [25, 27, 28, 32–34, 38] (including 1206 patients) and 6 studies [27, 28, 30, 32–34](including 2854 patients) reported lung infection and wound infection, respectively. Pooled analysis of fixed-effects models showed that transfusion strategy had no effect on lung infection (RR, 0.80; 95% CI, 0.54–1.18; *P* = 0.26) and wound infection (RR, 1.06; 95% CI, 0.64–1.19, *P* = 0.16) without heterogeneity (*I*^*2*^ = 0%) (Fig. 4B-C).

#### Cardiovascular events

**Fig. 5:**
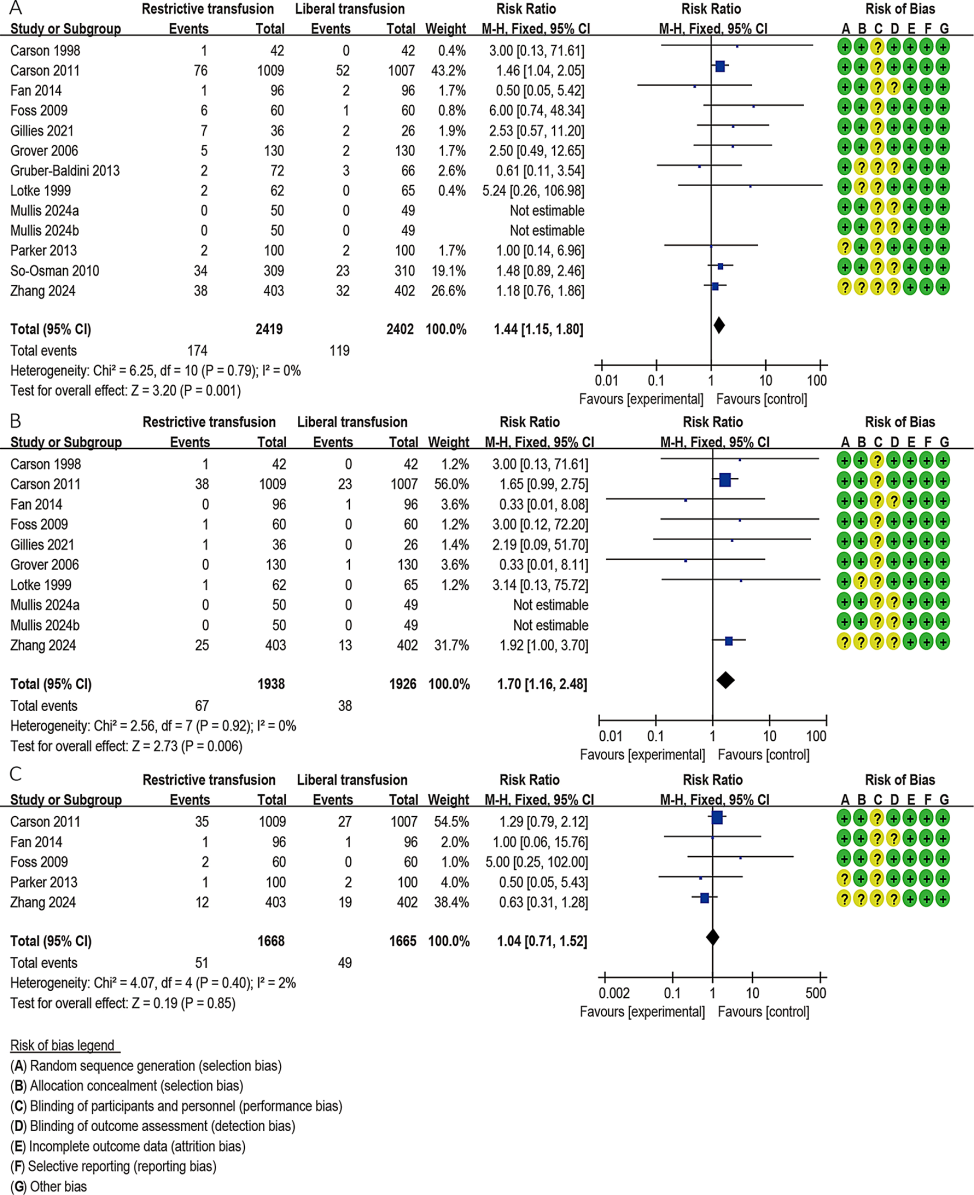
Forest plots depicting the comparison between the restrictive blood transfusion (RBT) group and liberal blood transfusion group: (**A**) Cardiovascular events; (**B**) Myocardial infarction; (**C**) Congestive heart failure

Thirteen studies involving 4821 patients reported cardiovascular events [17–19, 25–31, 33, 40]. The meta-analysis results of the fixed effects model showed that RBT can significantly increase cardiovascular events compared with the LBT group without heterogeneity (RR = 1.44; 95% CI: 1.15–1.80, *P* = 0.001; *I*^*2*^ = 0%) **(**Fig. 5A**)**. Among these studies, 10 studies [17–19, 25–27, 30, 33, 40](including 4864 patients) and 5 studies [28, 30, 32, 33, 40](including 3333 patients) reported on MI and CHF, respectively. Pooled analysis of the fixed-effects model showed that compared with LBT, RBT increased the risk of MI (RR = 1.70; 95% CI: 1.16–2.48, *P* = 0.006; *I*^*2*^ = 0%), but had no statistical significance for CHF (RR = 1.04; 95% CI: 0.71–1.52, *P* = 0.85; *I*^*2*^ = 2%) (Fig. 5B-C).

#### Thromboembolic events, cerebrovascular accidents, mortality, delirium, length of hospitalization, transfusion rates

Thromboembolic events (RR = 0.88; 95% CI: 0.33–2.31, *P* = 0.79; *I*^*2*^ = 0%), cerebrovascular accidents (RR = 0.50; 95% CI: 0.09–2.70, *P* = 0.42; *I*^*2*^ = 0%), mortality (≤ 30 days) (RR = 0.96; 95% CI: 0.87–1.06, *P* = 0.43; *I*^*2*^ = 14%), mortality (≥ 60 days) (RR = 0.94; 95% CI: 0.78–1.15, *P* = 0.57; *I*^*2*^ = 0%), delirium (RR = 1.33; 95% CI: 0.92–1.93, *P* = 0.13; *I*^*2*^ = 34%) and length of hospitalization (RR = -0.08; 95% CI: -0.22-0.06, *P* = 0.27; *I*^*2*^ = 57%) were not statistically different between the RBT and LBT groups (Supplementary Fig. 1–6). Analysis of data from 14 trials [17–19, 25, 27–34, 36, 37, 40]involving 5102 patients showed that transfusion rates in the RBT group were significantly lower than those in the LBT with high heterogeneity (RR = 0.70; 95% CI: 0.52–0.94, *P* = 0.02; *I*^*2*^ = 97%) (Supplementary Fig. 7).

### RBT (Threshold 7 to 8 g/dL) VS LBT (Threshold 9 to 10 g/dL)

According to clinical practice, the RBT threshold is usually 7 to 8 g/dL and the LBT threshold is 9 to 10 g/dL [1], and the two threshold ranges overlapped in included studies. Therefore, we only included nine studies within this common threshold for additional meta-analysis [17, 25, 27, 28, 30, 31, 33, 35, 40], and the results were consistent with the above results without significant difference. (Supplementary Fig. 8–20).

### Subgroup analysis results

Considering the characteristics of the included studies, the effect of transfusion strategy and the heterogeneity in the meta-analysis may be related to patient characteristics (patients at high risk for cardiovascular disease versus ordinary patients) and types of surgical procedures (total joint replacement or revision versus fracture repair). Therefore, we performed a subgroup analysis based on the above conditions and the results show that patient characteristics and types of surgical procedures do affect the meta-analysis results. The detailed results are shown in Table 3. Of note, in patients at high risk for cardiovascular disease, RBT increase the risk of MI and length of hospitalization compared with LBT. Additionally, for joint replacement or revision surgery, RBTs were associated with lower overall infections and shorter length of hospitalization compared with LBT groups. For fracture repair surgery, RBT increases the risk of MI.

**Table 3:** Subgroup analysis evaluating transfusion strategies in orthopedic patients

Variables	Outcome	Subgroup	Number of Experiments	Participants	Statistical Method	SMD [95% CI]/ RR [95% CI]	*P* Value	
Patient characteristics (patients at high risk for cardiovascular disease versus ordinary patients)								
	Overall infection		14	3207	Risk Ratio (M-H, Random, 95% CI)	0.82 [0.41, 1.64]	0.14	
		Patients at high risk for cardiovascular disease	2	943	Risk Ratio (M-H, Random, 95% CI)	0.71 [0.29, 1.75]	0.46	
		Ordinary patients	12	2264	Risk Ratio (M-H, Random, 95% CI)	0.81 [0.61, 1.07]	0.16	
	Cardiovascular events		13	4821	Risk Ratio (M-H, Fixed, 95% CI)	1.44 [1.15, 1.80]	0.001	
		Patients at high risk for cardiovascular disease	3	2959	Risk Ratio (M-H, Fixed, 95% CI)	1.33 [1.02, 1.74]	0.04	
		Ordinary patients	10	1862	Risk Ratio (M-H, Fixed, 95% CI)	1.74 [1.15, 2.63]	0.008	
	Myocardial infarction		10	3864	Risk Ratio (M-H, Fixed, 95% CI)	1.70 [1.16, 2.48]	0.006	
		Patients at high risk for cardiovascular disease	2	2821	Risk Ratio (M-H, Fixed, 95% CI)	1.75 [1.17, 2.61]	0.007	
		Ordinary patients	8	1043	Risk Ratio (M-H, Fixed, 95% CI)	1.34 [0.43, 4.23]	0.61	
	Congestive heart failure		5	3333	Risk Ratio (M-H, Fixed, 95% CI)	1.04 [0.71, 1.52]	0.85	
		Patients at high risk for cardiovascular disease	2	2821	Risk Ratio (M-H, Fixed, 95% CI)	1.02 [0.68, 1.52]	0.92	
		Ordinary patients	3	512	Risk Ratio (M-H, Fixed, 95% CI)	1.29 [0.32, 5.15]	0.72	
	Overall mortality (≤ 30 days)		11	6645	Risk Ratio (M-H, Fixed, 95% CI)	0.96 [0.87, 1.06]	0.43	
		Patients at high risk for cardiovascular disease	2	2821	Risk Ratio (M-H, Fixed, 95% CI)	0.78 [0.57, 1.08]	0.14	
		Ordinary patients	9	3824	Risk Ratio (M-H, Fixed, 95% CI)	0.99 [0.90, 1.10]	0.88	
	Overall mortality (≥ 60 days)		5	3367	Risk Ratio (M-H, Fixed, 95% CI)	0.94 [0.78, 1.15]	0.57	
		Patients at high risk for cardiovascular disease	2	2821	Risk Ratio (M-H, Fixed, 95% CI)	0.83 [0.64, 1.08]	0.17	
		Ordinary patients	3	546	Risk Ratio (M-H, Fixed, 95% CI)	1.16 [0.86, 1.56]	0.34	
	Transfusion rates		14	5102	Risk Ratio (M-H, Random, 95% CI)	0.70 [0.52, 0.94]	0.02	
		Patients at high risk for cardiovascular disease	3	2959	Risk Ratio (M-H, Random, 95% CI)	0.62 [0.33, 1.16]	0.14	
		Ordinary patients	11	2143	Risk Ratio (M-H, Random, 95% CI)	0.78 [0.55, 0.99]	0.04	
	Length of hospitalization		7	3296	Std. Mean Difference (IV, Fixed, 95% CI)	0.00 [-0.07, 0.07]	0.96	
		Patients at high risk for cardiovascular disease	2	2154	Std. Mean Difference (IV, Fixed, 95% CI)	0.08 [-0.01, 0.16]	0.07	
		Ordinary patients	5	1142	Std. Mean Difference (IV, Fixed, 95% CI)	-0.14 [-0.26, -0.03]	0.02	
**Surgical procedures (total joint replacement or revision versus fracture repair)**								
	Overall infection		14	3207	Risk Ratio (M-H, Random, 95% CI)	0.81 [0.61, 1.07]	0.14	
		Total joint replacement or revision	4	1137	Risk Ratio (M-H, Random, 95% CI)	0.63 [0.40, 0.97]	0.04	
		Fracture repair	10	2070	Risk Ratio (M-H, Random, 95% CI)	0.92 [0.70, 1.21]	0.54	
	Wound infection		6	2854	Risk Ratio (M-H, Fixed, 95% CI)	0.64 [0.34, 1.19]	0.16	
		Total joint replacement or revision	2	258	Risk Ratio (M-H, Fixed, 95% CI)	0.56 [0.12, 2.56]	0.45	
		Fracture repair	4	2596	Risk Ratio (M-H, Fixed, 95% CI)	0.66 [0.33, 1.30]	0.23	
	Lung infection		7	1206	Risk Ratio (M-H, Fixed, 95% CI)	0.80 [0.54, 1.18]	0.26	
		Total joint replacement or revision	2	258	Risk Ratio (M-H, Fixed, 95% CI)	0.47 [0.13, 1.63]	0.23	
		Fracture repair	5	948	Risk Ratio (M-H, Fixed, 95% CI)	0.86 [0.57, 1.30]	0.47	
	Cardiovascular events		13	4821	Risk Ratio (M-H, Fixed, 95% CI)	1.44 [1.15, 1.80]	0.001	
		Total joint replacement or revision	3	1006	Risk Ratio (M-H, Fixed, 95% CI)	1.64 [1.02, 2.62]	0.04	
		Fracture repair	10	3815	Risk Ratio (M-H, Fixed, 95% CI)	1.39 [1.08, 1.79]	0.01	
	Myocardial infarction		10	3864	Risk Ratio (M-H, Fixed, 95% CI)	1.70 [1.16, 2.48]	0.006	
		Total joint replacement or revision	3	579	Risk Ratio (M-H, Fixed, 95% CI)	0.73 [0.15, 3.63]	0.70	
		Fracture repair	7	3285	Risk Ratio (M-H, Fixed, 95% CI)	1.79 [1.21, 2.65]	0.004	
	Overall mortality (≤ 30 days)		11	6645	Risk Ratio (M-H, Fixed, 95% CI)	0.96 [0.87, 1.06]	0.43	
		Total joint replacement or revision	2	879	Risk Ratio (M-H, Fixed, 95% CI)	0.43 [0.06, 2.89]	0.39	
		Fracture repair	9	5766	Risk Ratio (M-H, Fixed, 95% CI)	0.96 [0.87, 1.06]	0.47	
	Transfusion rates		14	5102	Risk Ratio (M-H, Random, 95% CI)	0.70 [0.52, 0.94]	0.02	
		Total joint replacement or revision	4	1137	Risk Ratio (M-H, Random, 95% CI)	0.89 [0.70, 1.12]	0.31	
		Fracture repair	10	3965	Risk Ratio (M-H, Random, 95% CI)	0.63 [0.43, 0.94]	0.02	
	Delirium		5	644	Risk Ratio (M-H, Fixed, 95% CI)	1.33 [0.92, 1.93]	0.13	
		Total joint replacement or revision	2	319	Risk Ratio (M-H, Fixed, 95% CI)	1.13 [0.69, 1.86]	0.62	
		Fracture repair	3	325	Risk Ratio (M-H, Fixed, 95% CI)	1.62 [0.92, 2.86]	0.10	
	Length of hospitalization		7	3296	Std. Mean Difference (IV, Fixed, 95% CI)	0.00 [-0.07, 0.07]	0.96	
		Total joint replacement or revision	3	938	Std. Mean Difference (IV, Fixed, 95% CI)	-0.16 [-0.29, -0.03]	0.01	
		Fracture repair	4	2358	Std. Mean Difference (IV, Fixed, 95% CI)	0.07 [-0.02, 0.15]	0.11	

Abbreviation: RR, risk ratio; SMD, standardized mean difference

### Sensitivity analysis and publication bias

The results of sensitivity analysis and publication bias are shown in Table 4. The results obtained by the sensitivity analysis were basically consistent with the results of all included studies, indicating that our results are stable (See Supplementary Fig. 21–24). However, only overall infection, lung infection and cerebrovascular accident did not show publication bias in Egger’s test (*p* < 0.05). The remaining results were analyzed using the trim and fill method (Supplementary Table 2). Wound infection, CHF, delirium, and length of hospitalization were not trimmed, and the data in the funnel plot remained unchanged, indicating that there was no significant publication bias for these outcomes.

**Table 4:** Sensitivity and publication bias analysis

Outcome	SMD/RR fluctuation	95% CI fluctuation	Publication bias (*P* value)	Pooling model
Overall infection	0.69–0.89	0.47–1.15	0.001	Random (I-V heterogeneity)
Lung infection	0.50–0.92	0.22–1.38	0.013	Fixed Inverse Variance
Wound infection	0.57–0.78	0.29–2.12	0.96	Fixed Inverse Variance
Cardiovascular Events	1.39–1.51	1.02–1.96	0.407	Fixed Inverse Variance
Myocardial infarction	1.61–1.80	1.00-3.22	0.652	Fixed Inverse Variance
Congestive heart failure	0.70–1.28	0.37–1.57	0.981	Fixed Inverse Variance
Thromboembolic Events	0.76–1.18	0.22–3.79	0.518	Fixed Inverse Variance
Cerebrovascular Accidents	0.33–0.63	0.04–5.02	0.019	Fixed Inverse Variance
Mortality (≤ 30 days)	0.94-1.00	0.76–1.3	0.31	Fixed Inverse Variance
Mortality (≥ 60 days)	0.87–1.02	0.70–1.31	0.574	Fixed Inverse Variance
Transfusion rates	0.67–0.73	0.51–0.97	0.883	Random (I-V heterogeneity)
Delirium	1.05–1.80	0.67–3.1	0.758	Fixed Inverse Variance
Length of hospitalization	(-0.13)-(0.00)	(-0.26)-(0.09)	0.085	Random (I-V heterogeneity)

Abbreviation: RR, risk ratio; SMD, standardized mean difference

### Trial sequential analysis

**Fig. 6:**
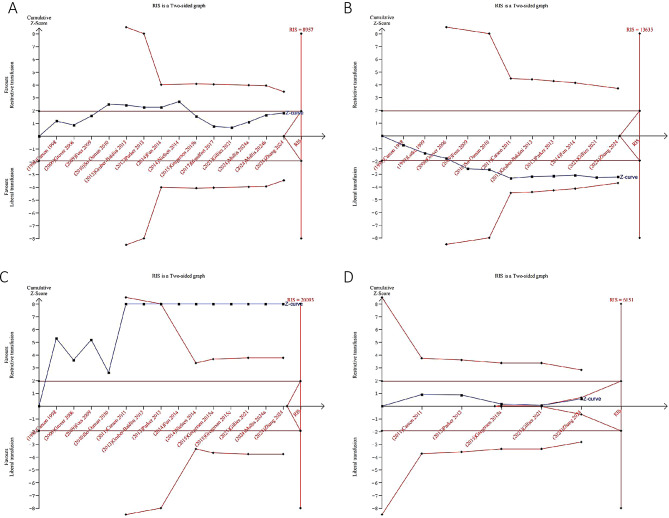
Trial sequential analysis. A required information size was calculated based on using α = 0.05 (two-sided), β = 0.20 (power 80%), and an anticipated relative risk reduction (RRR) of 20%. (**A**) Overall infection; (**B**) Cardiovascular events; (**C**) Transfusion rates; (**D**) Mortality (≥ 60 days)

TSA can assess whether the cumulative effect of studies included in a meta-analysis is supported by sufficient data. TSA results indicate that there is sufficient data to draw definite conclusions about the impact of transfusion strategies on transfusion rates and overall mortality (≥ 60 days). However, the evidence for efficacy for the remaining outcomes analyzed (including infection, cardiovascular events, thromboembolic events, cerebrovascular accidents, mortality, delirium, length of hospitalization) was inconclusive. In other words, the currently accumulated number of clinical trial participants is too small, which may lead to insufficient conclusions in this meta-analysis. Therefore, more high-quality clinical trials with large populations can be conducted in the future to further verify these conclusions (Fig. 6 and Supplementary Fig. 25).

## Discussion

### Principal findings

This meta-analysis included 19 studies involving a total of 7833 patients. The risk of bias in the included studies was low, and the overall analysis results were of moderate quality of evidence. The results show that compared with the LBT strategy, the RBT strategy can increase the occurrence of cardiovascular events, which mainly increases MI rather than CHF. In addition, the blood transfusion rate in the RBT group was higher than that in the LBT group, but there was no difference in the impact of RBT strategies and LBT strategies on infection, thromboembolic events, cerebrovascular accidents, delirium, length of hospitalization and mortality. The results of the subgroup analysis showed that whether the patient was at risk for cardiovascular disease and the type of surgery were important factors affecting the adverse reactions of the blood transfusion strategy. The TSA results showed that except for the results of transfusion rates and overall mortality (≥ 60 days), which were supported by sufficient evidence, the other results were inconclusive due to the small sample size.

Blood transfusions are associated with numerous complications, such as transfusion-related lung injury, cardiac overload, immune responses, and infectious diseases [41–43]. Blood transfusions have been shown to be strongly associated with recurrent thrombosis and death in patients with acute coronary syndrome and/or MI [44, 45]. In the clinical studies we included, we found that most of the patients undergoing orthopedic surgery are elderly and cannot tolerate a hypoxic environment. Excessive blood transfusion or excessive fluid delivery can easily lead to fluid circulation overload and increase the burden on the heart. These may increase the probability of adverse transfusion reactions.

Transfusion-associated circulatory overload (TACO) is the most common pulmonary complication of transfusion and the leading cause of transfusion-related death [1]. Among our included studies, one study reported one case of TACO in the restricted group [17], and another reported five cases of TACO (but the transfusion method was unknown [29].

### Comparison with existing literature

In 2015, Brunskill et al. [11] conducted a meta-analysis to evaluate the benefits and harms of RBC transfusion strategies in patients undergoing hip fracture surgery. They included six trials (2722 participants) that showed a reduced risk of MI but no difference in thromboembolism, wound infection, CHF and mortality in LBT groups compared with RBT groups, which were also consistent with our findings. Wang et al. [16] and Mitchell et al. [14] reported no statistically significant relationship between blood transfusion strategy and infections in orthopedic patients, but Teng’s study found that a RBT strategy could significantly reduce infections [15]. Mao et al. [13] found that the blood transfusion strategy did not affect the incidence of MI. These differences may be due to the small number of included studies and the different effect models used. Different from previously published meta-analyses on the same topic, our article systematically screened and updated the literature and included more RCTs. The most recent meta that has been published so far was searched in July 2019, and only studied the relationship between blood transfusion and infection rates [16]. Most existing studies have only searched for hip and knee surgeries. We searched for all orthopedic-related surgeries and also studied the relationship between transfusion strategies and various outcome measures. We also analyzed the relationship between different subgroups. Therefore, our research is a necessary update and improvement of previous research.

### Strengths of this meta-analysis

First, this article is a comprehensive updated meta-analysis. It conducted a comprehensive and detailed literature search and screening, included a larger number of studies and participants, and covered studies published from 1998 to 2024 (4/19 of them were published in the last 3 years). Second, the low heterogeneity of the meta-analysis, the good consistency of the sensitivity analysis, the low risk of bias and the moderate quality of evidence all suggest that our analysis results are stable and reliable. Third, compared with the recently published meta-analysis on the same topic, we updated RCTs in the past 5 years and included multiple outcomes such as cardiovascular events, thromboembolic events, mortality, delirium, length of hospitalization, etc. This has important implications for updating the latest research results and comprehensively comparing the impact of transfusion strategies on orthopedic patients. Fourth, we used TSA to evaluate the adequacy of the results, which has not been used in previously published articles on the same topic. Fifth, the studies included in this article are of higher quality evidence from RCTs, and compared with cross-sectional studies and cohort studies, some confounding factors have been controlled.

### Limitations

Our study has several limitations. Since the existing RCTs do not provide detailed individual patient data and blood transfusion conditions, we are unable to analyze the impact of blood transfusion strategies on different factors such as patient age, underlying diseases, gender, and disease type. The amount, type, suspension, and leucocyte information of RBCs used for transfusion were not clearly reported, and these differences in transfusion strategies may also be a source of part of the heterogeneity. Egger test and trim-and-fill analysis showed that some outcomes had a certain publication bias, which may be related to the small number of RCTs reporting this outcome. In addition, the blood transfusion process may be accompanied by the use of some drugs or interventions to reduce blood loss, RBC requirements and infection rates, such as perioperative iron and erythropoietin supplementation, which our article did not consider.

### Implications for clinical practice and research

Our analysis found that RBT reduce transfusion rates but may increase the risk of cardiovascular events (mainly MI), especially in patients at high risk for cardiovascular disease. Furthermore, RBT have varying effects in different orthopedic surgeries. Therefore, we need to be cautious about blood transfusions in clinical practice, and comprehensively consider patient characteristics and surgery types when making decisions. TACO and transfusion-related acute lung injury (TRALI), the main cause of death among blood transfusion associated complications, refer to acute respiratory distress syndrome occurring within 6 h after blood transfusion [1]. These two are the dominant factors in weighing the risks of transfusion, but they are rarely reported in the included studies and deserve attention in future studies. In the future, we also hope that more research will be done to analyze the mechanism of adverse reactions caused by blood transfusion strategies in order to come up with countermeasures.

## Conclusion

This meta-analysis compared the effects of blood transfusion strategies in orthopedic patients using outcome measures such as infection, cardiovascular events, cerebrovascular accidents, and mortality. The overall results indicate that the RBT strategy increases the risk of cardiovascular events (mainly MI) and reduces transfusion rates, but has no statistically significant effect on other outcome measures. The patient’s cardiovascular disease risk and type of surgery also influence the outcome of the transfusion strategy. Potential differences between RBT and LBT strategies remain to be explored in larger, higher-quality clinical trials.

## Electronic supplementary material

Below is the link to the electronic supplementary material.

Supplementary Material 1

## Data Availability

No datasets were generated or analysed during the current study.

## Declarations

### PROSPERO registration no

CRD42024604868.
