# Supplementary material for: Restrictive versus Liberal blood transfusion strategies for patients undergoing orthopedic surgery: a meta-analysis of randomised trials with trial sequential analysis
Source: J Orthop Surg Res. 2025 May 24;20:513. doi: 10.1186/s13018-025-05883-0 (PMC12102801; doi:10.1186/s13018-025-05883-0)
Supplement: Supplementary file 1 — Supplementary Material 1 [file 13018_2025_5883_MOESM1_ESM.docx]

**1. Literature search strategy**

1.1 Search strategies in Pubmed

Last searched October 19, 2024

| No | Query | Results |
| --- | --- | --- |
| #1 | Orthopedic Procedures[mh] OR Orthopedics[mh] OR Fracture, Bone[mh] OR Arthroplasty, Replacement[mh] OR orthopedic procedure[tiab] OR orthopedic procedures[tiab] OR Orthopedic Surgery[tiab] OR Orthopedic Surgeries[tiab] OR Orthopedic Surgical Procedure[tiab] OR Orthopedic Surgical Procedures[tiab] OR fracture[tiab] OR Broken Bones[tiab] OR Joint Replacement[tiab] OR Joint Replacements[tiab] OR Replacement Arthroplasties[tiab] OR Replacement Arthroplasty[tiab] OR Joint Prosthesis Implantation[tiab] OR Joint Prosthesis Implantations[tiab] OR Total Joint Replacements[tiab] OR Total Joint Replacement[tiab] OR orthopaedic[tiab] OR orthopedic[tiab] OR orthopaedics[tiab] OR orthopedics[tiab] OR prosthesis[tiab] OR prosthetic[tiab] OR arthroplasty[tiab] OR arthroplasties[tiab] OR arthrodesis[tiab] OR arthroscopy[tiab] OR diskectomy[tiab] OR osteotomy[tiab] OR tenodesis[tiab] | 825069 |
| #2 | Blood Transfusion[mh] OR ((transfusion[tiab] OR infusion[tiab] OR hypertransfusion[tiab] OR retransfusion[tiab]) AND (blood[tiab] OR erythrocyte[tiab] OR red cell[tiab] OR red blood cell[tiab] OR whole blood[tiab])) | 206197 |
| #3 | (randomized controlled trial[pt] OR controlled clinical trial[pt] OR randomized[tiab] OR placebo[tiab] OR randomly[tiab] OR trial[tiab]) NOT (animals[mh] NOT humans[mh]) | 1643786 |
| #4 | #1 AND #2 AND #3 | 1867 |

.

2.2. Search strategies in Web of Science

Last searched October 19, 2024

| No. | Query | Results |
| --- | --- | --- |
| #1 | TS=(orthopedic procedure OR orthopedic procedures OR Orthopedic Surgery OR Orthopedic Surgeries OR Orthopedic Surgical Procedure OR Orthopedic Surgical Procedures OR fracture OR Broken Bones OR Joint Replacement OR Joint Replacements OR Replacement Arthroplasties OR Replacement Arthroplasty OR Joint Prosthesis Implantation OR Joint Prosthesis Implantations OR Total Joint Replacements OR Total Joint Replacement OR orthopaedic OR orthopedic OR orthopaedics OR orthopedics OR prosthesis OR prosthetic OR arthroplasty OR arthroplasties OR arthrodesis OR arthroscopy OR diskectomy OR osteotomy OR tenodesis) | 834816 |
| #2 | TS=(Blood Transfusion OR ((transfusion OR infusion OR hypertransfusion OR retransfusion) AND (blood OR erythrocyte OR red cell OR red blood cell OR whole blood))) | 122159 |
| #3 | TS=(randomized controlled trial OR controlled clinical trial OR randomized OR placebo OR randomly) | 1579451 |
| #4 | #3 AND #2 AND #1 | 1836 |

2.3. Search strategies in the Cochrane Central Register of Controlled Trials

Last searched October 19, 2024

| No. | Query | Results |
| --- | --- | --- |
| #1 | MeSH descriptor: [Orthopedic Procedures] explode all trees | 20197 |
| #2 | MeSH descriptor: [Orthopedics] explode all trees | 580 |
| #3 | MeSH descriptor: [Fractures, Bone] explode all trees | 9656 |
| #4 | MeSH descriptor: [Arthroplasty, Replacement] explode all trees | 6997 |
| #5 | (orthopedic procedure OR orthopedic procedures OR Orthopedic Surgery OR Orthopedic Surgeries OR Orthopedic Surgical Procedure OR Orthopedic Surgical Procedures OR fracture OR Broken Bones OR Joint Replacement OR Joint Replacements OR Replacement Arthroplasties OR Replacement Arthroplasty OR Joint Prosthesis Implantation OR Joint Prosthesis Implantations OR Total Joint Replacements OR Total Joint Replacement OR orthopaedic OR orthopedic OR orthopaedics OR orthopedics OR prosthesis OR prosthetic OR arthroplasty OR arthroplasties OR arthrodesis OR arthroscopy OR diskectomy OR osteotomy OR tenodesis):ti,ab,kw | 72099 |
| #6 | #1 OR #2 OR #3 OR #4 OR #5 | 76958 |
| #7 | MeSH descriptor: [Blood Transfusion] explode all trees | 4815 |
| #8 | (Blood Transfusion OR ((transfusion OR infusion OR hypertransfusion OR retransfusion) AND (blood OR erythrocyte OR red cell OR red blood cell OR whole blood))):ti,ab,kw | 46812 |
| #9 | #7 OR #8 | 47225 |
| #10 | (randomized controlled trial OR controlled clinical trial OR randomized OR placebo OR randomly OR trial):ti,ab,kw | 1561100 |
| #11 | #6 AND #9 AND #10 | 2738 |

2.4. Search strategies in EMBASE (Embase.com)

Last searched October 19, 2024

| No. | Query | Results |
| --- | --- | --- |
|  | 'orthopedic procedure':ab,ti OR 'orthopedic procedures':ab,ti OR 'orthopedic surgery':ab,ti OR 'orthopedic surgeries':ab,ti OR 'orthopedic surgical procedure':ab,ti OR 'orthopedic surgical procedures':ab,ti OR fracture:ab,ti OR 'broken bones':ab,ti OR 'joint replacement':ab,ti OR 'joint replacements':ab,ti OR 'replacement arthroplasties':ab,ti OR 'replacement arthroplasty':ab,ti OR 'joint prosthesis implantation':ab,ti OR 'joint prosthesis implantations':ab,ti OR 'total joint replacements':ab,ti OR 'total joint replacement':ab,ti OR orthopaedic:ab,ti OR orthopedic:ab,ti OR orthopaedics:ab,ti OR orthopedics:ab,ti OR prosthesis:ab,ti OR prosthetic:ab,ti OR arthroplasty:ab,ti OR arthroplasties:ab,ti OR arthrodesis:ab,ti OR arthroscopy:ab,ti OR diskectomy:ab,ti OR osteotomy:ab,ti OR tenodesis:ab,ti OR 'orthopedic surgery'/exp OR 'orthopedics'/exp OR 'fracture'/exp OR 'replacement arthroplasty'/exp | 1345272 |
|  | (transfusion:ab,ti OR infusion:ab,ti OR hypertransfusion:ab,ti OR retransfusion:ab,ti) AND (blood:ab,ti OR erythrocyte:ab,ti OR 'red cell':ab,ti OR 'red blood cell':ab,ti OR 'whole blood':ab,ti) OR 'blood transfusion'/exp | 381656 |
| #3 | 'crossover procedure':de OR 'double-blind procedure':de OR 'randomized controlled trial':de OR 'single-blind procedure':de OR random*:de,ab,ti OR factorial*:de,ab,ti OR crossover*:de,ab,ti OR ((cross NEXT/1 over*):de,ab,ti) OR placebo*:de,ab,ti OR ((doubl* NEAR/1 blind*):de,ab,ti) OR ((singl* NEAR/1 blind*):de,ab,ti) OR assign*:de,ab,ti OR allocat*:de,ab,ti OR volunteer*:de,ab,ti | 3415738 |
|  | #1 AND #2 AND #3 | 3655 |

2.5. Search strategies in clinicaltrials.gov

Last searched October 19, 2024

Condition/disease：orthopedic procedure OR orthopedic procedures OR Orthopedic Surgery OR Orthopedic Surgeries OR Orthopedic Surgical Procedure OR Orthopedic Surgical Procedures OR fracture OR Broken Bones OR Joint Replacement OR Joint Replacements OR Replacement Arthroplasties OR Replacement Arthroplasty OR Joint Prosthesis Implantation OR Joint Prosthesis Implantations OR Total Joint Replacements OR Total Joint Replacement OR orthopaedic OR orthopedic OR orthopaedics OR orthopedics OR prosthesis OR prosthetic OR arthroplasty OR arthroplasties OR arthrodesis OR arthroscopy OR diskectomy OR osteotomy OR tenodesis

Intervention/treatment

Blood Transfusion OR ((transfusion OR infusion OR hypertransfusion OR retransfusion) AND (blood OR erythrocyte OR red cell OR red blood cell OR whole blood))

Study Status： All study

Study Results： With results

Results ：25

**2. Reasons for excluding studies after reading the full text**

| **Study** | **Reasons for exclusion** | **Reference** |
| --- | --- | --- |
| Atay 2010 | Intervention did not meet inclusion criteria. | (1) |
| Beaupre 2018 | Intervention did not meet inclusion criteria. | (2) |
| Blumberg 2014 | Not randomized controlled trial | (3) |
| Blandfort 2015 | Conference abstract | (4) |
| Carson 2011 | Conference abstract | (5) |
| Carson 2014 | Conference abstract | (6) |
| Cuenca 2007 | Intervention did not meet inclusion criteria. | (7) |
| Fernández-Cortiñas 2023 | Not randomized controlled trial | (8) |
| Gregersen 2013 | Conference abstract | (9) |
| Gregersen 2014 | Conference abstract | (10) |
| Gregersen 2016 | Not randomized controlled trial | (11) |
| Grover 2004 | Conference abstract | (12) |
| Gruber-Baldini 2010 | Conference abstract | (13) |
| Gruber-Baldini 2011 | Conference abstract | (14) |
| Gupta 2018 | Not randomized controlled trial | (15) |
| Møller 2019 | Not orthopedic surgery. | (16) |
| Muñoz 2009 | Not randomized controlled trial | (17) |
| Naylor 2010 | Not randomized controlled trial | (18) |
| Nielsen 2012 | No outcome data provided | (19) |
| Purvis 2017 | Conference abstract | (20) |
| Qiu 2021 | Intervention did not meet inclusion criteria. | (21) |
| So-Osman 2016 | Conference abstract | (22) |
| So-Osman 2013 | No outcome data provided | (23) |
| So-Osman 2014 | Intervention did not meet inclusion criteria. | (24) |
| So-Osman 2008 | Conference abstract | (25) |
| So-Osman 2011a | Conference abstract | (26) |
| So-Osman 2011b | No outcome data provided | (27) |
| Vamvakas 1995 | Not randomized controlled trial | (28) |
| Viberg 2018 | Not randomized controlled trial | (29) |
| Vichinsky 1995 | Not orthopedic surgery. | (30) |
| Yu 2019 | Not randomized controlled trial | (31) |
| Zhang 2024 | Not randomized controlled trial | (32) |

**Reference：**

1. Atay EF, Güven M, Altıntaş F, Kadıoğlu B, Ceviz E, Ipek S. Allogeneic blood transfusion decreases with postoperative autotransfusion in hip and knee arthroplasty. Acta Orthop Traumatol Turc. 2010;44(4):306-12.

2. Beaupre LA, Wai EK, Hoover DR, Noveck H, Roffey DM, Cook DR, et al. A comparison of outcomes between Canada and the United States in patients recovering from hip fracture repair: secondary analysis of the FOCUS trial. Int J Qual Health Care. 2018;30(2):97-103.

3. Blumberg N, Rogers M. Leukoreduction and restrictive red blood cell (RBC) transfusion minimize healthcare-associated infections after orthopedic surgery. Transfusion. 2014;54:187A.

4. Blandfort S, Damsgaard EM, Gregersen M. Blood transfusion strategy and risk of postoperative delirium in nursing homes residents with hip fracture. European Geriatric Medicine. 2015;6:S77.

5. Carson J. FOCUS: Transfusion triggers after fractured hip repair. Transfusion Medicine. 2011;21:14.

6. Carson JL, Sieber F, Hoover DR, Noveck H, Chaitman BR, Beaupre L, et al. A randomized clinical trial of liberal versus restrictive transfusion strategy evaluating long term survival and cause of death: Results from the FOCUS Trial. Blood. 2014;124(21).

7. Cuenca J, García-Erce JA, Martínez F, Cardona R, Pérez-Serrano L, Muñoz M. Preoperative haematinics and transfusion protocol reduce the need for transfusion after total knee replacement. Int J Surg. 2007;5(2):89-94.

8. Fernández-Cortiñas AB, Seoane-Pillado T, Marco Martínez F. Blood transfusion and surgical treatment increase mortality in patient with proximal humeral fractures. Injury. 2023;54 Suppl 7:111091.

9. Gregersen M, Borris LC, Damsgaard EM. A liberal blood transfusion strategy improves survival in nursing home residents with hip fracture. European Geriatric Medicine. 2013;4:S106.

10. Gregersen MG, Damsgaard EM, Borris LC. Blood transfusion strategies in recovery from physical disability and overall quality of life in frail elderly with hip fracture. European Geriatric Medicine. 2014;5:S228.

11. Gregersen M. Postoperative red blood cell transfusion strategy in frail anemic elderly with hip fracture: A randomized controlled trial. Danish Medical Journal. 2016;63(4).

12. Grover M, Talwalkar A, Casbard A, Boralessa H, Contreras M, Boralessa H, et al. Blood transfusion threshold and silent myocardial ischaemia after lower limb arthroplasty: a randomized controlled trial of transfusion strategy. British Journal of Anaesthesia. 2004;92(2):306-.

13. Gruber-Baldini A, Marcantonio ER, Orwig D, Magaziner J, Terrin M, Carson J, et al. FOCUS cognitive ancillary study: Randomized clinical trial of blood transfusion thresholds on delirium severity. Journal of the American Geriatrics Society. 2010;58:S86.

14. Gruber-Baldini AL, Marcatonio E, Orwig D, Magaziner J, Terrin M, Carson J. Blood transfusion and delirium at 45-days post-hip fracture: Longterm outcomes from the focus cognitive ancillary study. Journal of the American Geriatrics Society. 2011;59:S113.

15. Gupta PB, DeMario VM, Amin RM, Gehrie EA, Goel R, Ken Lee KH, et al. Patient blood management program improves blood use and clinical outcomes in orthopedic surgery. Anesthesiology. 2018;129(6):1082-91.

16. Møller A, Nielsen H, Wetterslev J, Pedersen O, Hellemann D, Winkel P, et al. Low vs. high hemoglobin trigger for transfusion in vascular surgery (TV): A randomized clinical feasibility trial (the TV trial). Vox Sanguinis. 2019;114:45.

17. Muñoz M, García-Erce JA, Villar I, Thomas D. Blood conservation strategies in major orthopaedic surgery: Efficacy, safety and European regulations. Vox Sanguinis. 2009;96(1):1-13.

18. Naylor JM, Adie S, Fransen M, Dietsch S, Harris I. Endorsing single-unit transfusion combined with a restrictive haemoglobin transfusion threshold after knee arthroplasty. Quality & Safety in Health Care. 2010;19(3):239-43.

19. Nielsen K, Dahl B, Johansson PI, Henneberg SW, Rasmussen LS. Intraoperative transfusion threshold and tissue oxygenation: A randomised trial. Transfusion Medicine. 2012;22(6):418-25.

20. Purvis TE, Goodwin CR, De La Garza-Ramos R, Ahmed AK, Lafage V, Neuman BJ, et al. Economic impact and clinical outcomes of liberal blood transfusion in spine surgery. Global Spine Journal. 2017;7(2):48S.

21. Qiu X, Tan Z, Tang W, Ye H, Lu X. Effects of controlled hypotension with restrictive transfusion on intraoperative blood loss and systemic oxygen metabolism in elderly patients who underwent lumbar fusion. Trials. 2021;22(1).

22. So-Osman C, Balradj J, Koopman-Van Gemert AWMM, Onstenk R, Van Den Hout WB, Brand R, et al. Patient blood management in elective orthopaedic hip-and knee replacement surgery-a report on clinical outcome. Vox Sanguinis. 2016;111:282.

23. So-Osman C, Nelissen R, Brand R, Faber F, Te Slaa R, Stiggelbout A, et al. The impact of a restrictive transfusion trigger on post-operative complication rate and well-being following elective orthopaedic surgery: a post-hoc analysis of a randomised study. Blood Transfusion. 2013;11(2):289-95.

24. So-Osman C, Nelissen R, Koopman-van Gemert A, Kluyver E, Pöll RG, Onstenk R, et al. Patient Blood Management in Elective Total Hip-and Knee-replacement Surgery ( Part 2). Anesthesiology. 2014;120(4):852-60.

25. So-Osman C, Nelissen R, Te Slaa R, Coene L, Brand R, Brand A. A randomized comparison of transfusion triggers in elective orthopedic surgery using leukocyte-depleted red blood cells. Transfusion alternatives in transfusion medicine. 2008;10(Suppl 1):30, Abstract No. P1.

26. So-Osman C, Nelissen RGHH, Koopman-van Gemert A, Kluyver E, Pöll R, Onstenk R, et al. A randomised controlled trial on erythropoietin and blood salvage as transfusion alternatives in orthopaedic surgery using a restrictive transfusion policy. Transfusion Alternatives in Transfusion Medicine. 2011;12(1):25-6.

27. So-Osman C, Nelissen R, Brand R, Brand A, Stiggelbout AM. Postoperative anemia after joint replacement surgery is not related to quality of life during the first two weeks postoperatively. Transfusion. 2011;51(1):71-81.

28. Vamvakas EC, Moore SB, Cabanela M. Blood transfusion and septic complications after hip replacement surgery. Transfusion. 1995;35(2):150-6.

29. Viberg B, Gundtoft PH, Schonnemann J, Pedersen L, Andersen LR, Titlestad K, et al. Introduction of national guidelines for restrictive blood transfusion threshold for hip fracture patients-a conse

cutive cohort study based on complete follow-up in national databases. Journal of Orthopaedic Surgery and Research. 2018;13.

30. Vichinsky EP, Haberkern CM, Neumayr L, Earles AN, Black D, Koshy M, et al. A comparison of conservative and aggressive transfusion regimens in the perioperative management of sickle cell disease. The Preoperative Transfusion in Sickle Cell Disease Study Group. N Engl J Med. 1995;333(4):206-13.

31. Yu XC, Wang ZX, Wang YP, Huang YG, Xin SJ, Sun H, et al. Cost-effectiveness comparison of routine transfusion with restrictive and liberal transfusion strategies for surgical patients in China. Vox Sanguinis. 2019;114(7):721-39.

32. Zhang YP, Dai J, Tang XM, Ma J. Establishment of a predictive model for blood transfusion after femoral head replacement in elderly patients. Joint Diseases and Related Surgery. 2024;35(3):538-45.

**3.Supplementary figures**


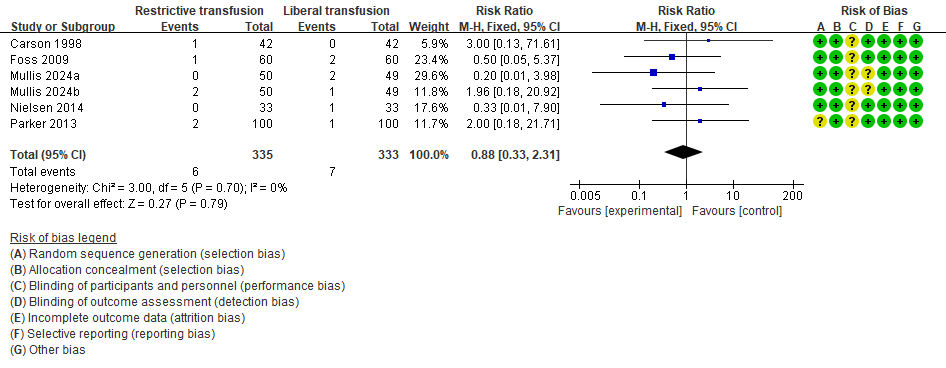


Supplementary figure 1 Forest plots depicting the comparison between the restrictive blood transfusion group and liberal blood transfusion group: Thromboembolic events.


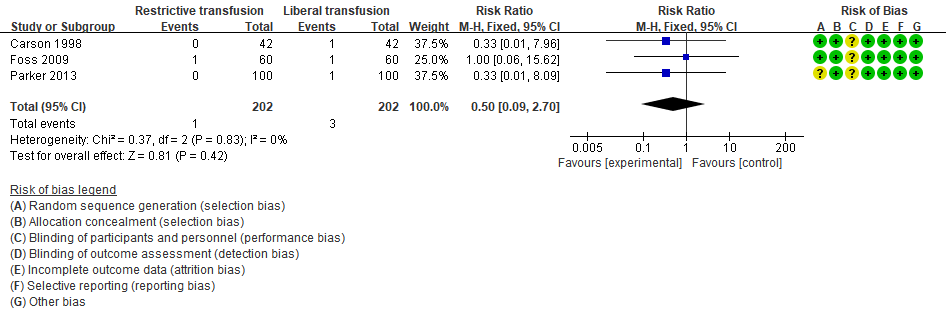


Supplementary figure 2 Forest plots depicting the comparison between the restrictive blood transfusion group and liberal blood transfusion group: Cerebrovascular accidents.


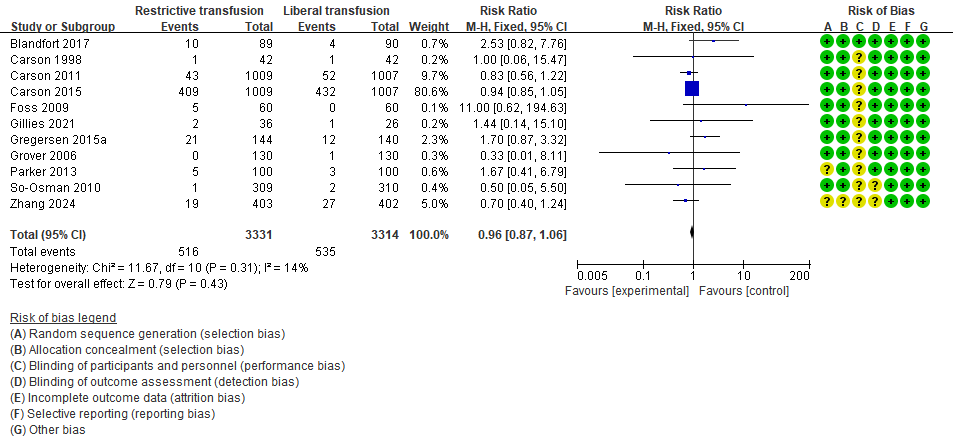


Supplementary figure 3 Forest plots depicting the comparison between the restrictive blood transfusion group and liberal blood transfusion group: Mortality (≤30 days).


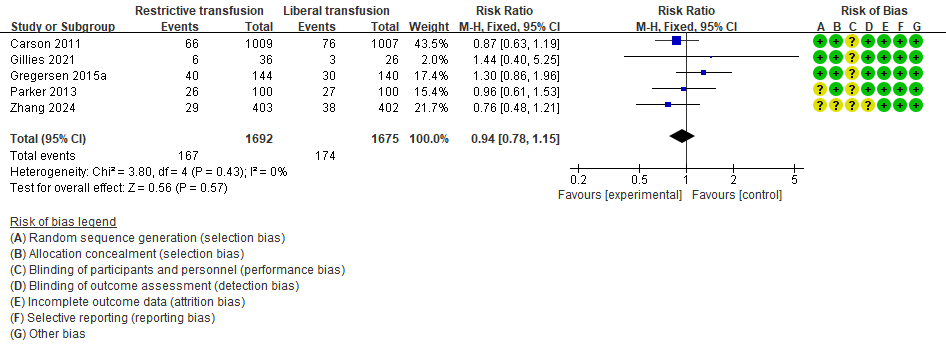


Supplementary figure 4 Forest plots depicting the comparison between the restrictive blood transfusion group and liberal blood transfusion group: Mortality (≥60 days).


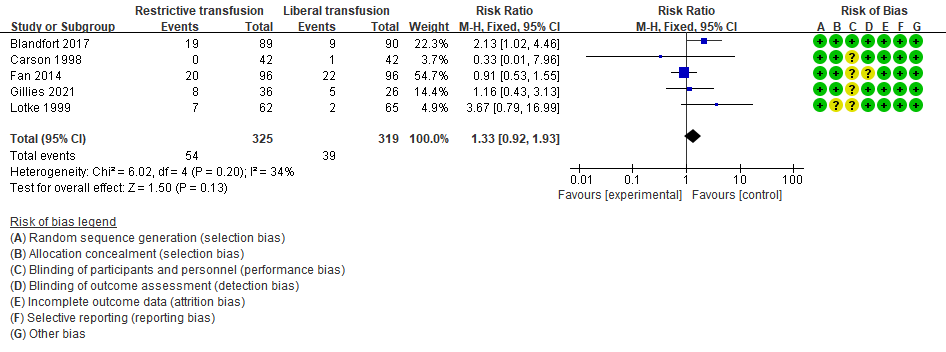


Supplementary figure 5 Forest plots depicting the comparison between the restrictive blood transfusion group and liberal blood transfusion group: Delirium.


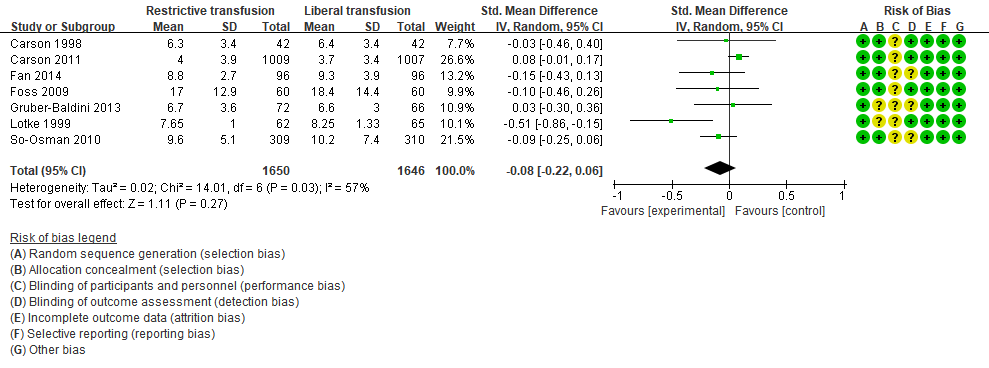


Supplementary figure 6 Forest plots depicting the comparison between the restrictive blood transfusion group and liberal blood transfusion group: Length of hospitalization.


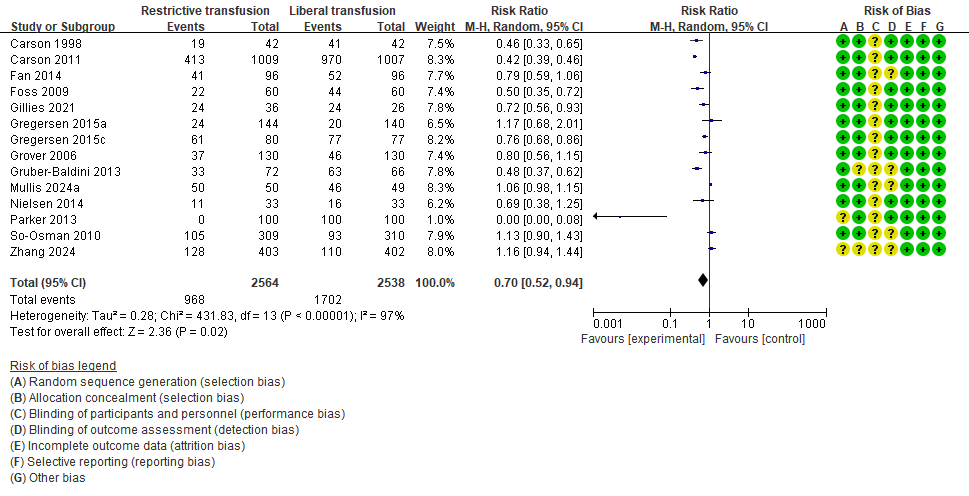


Supplementary figure 7 Forest plots depicting the comparison between the restrictive blood transfusion group and liberal blood transfusion group: Transfusion rates.


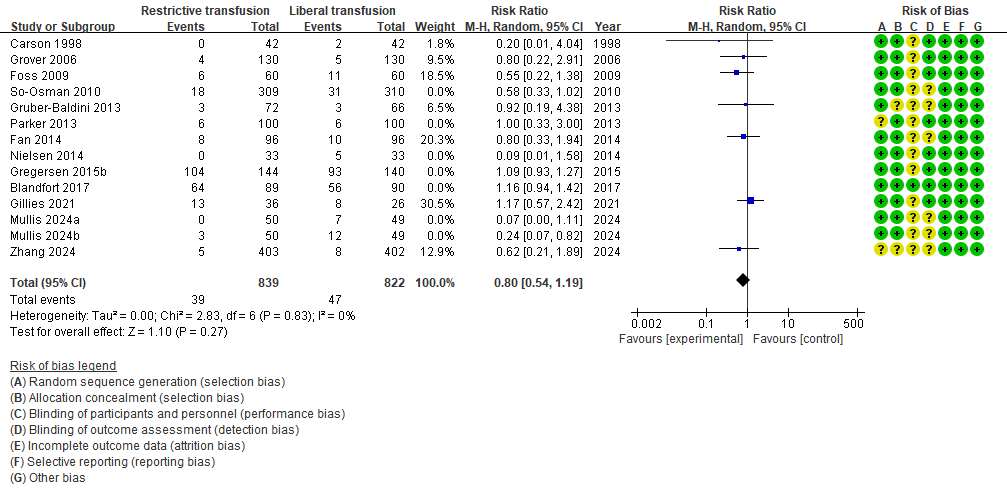


Supplementary figure 8 Forest plots depicting the comparison between the restrictive blood transfusion group (Threshold 7 to 8 g/dL) and liberal blood transfusion group (Threshold 9 to 10 g/dL): Overall infection.


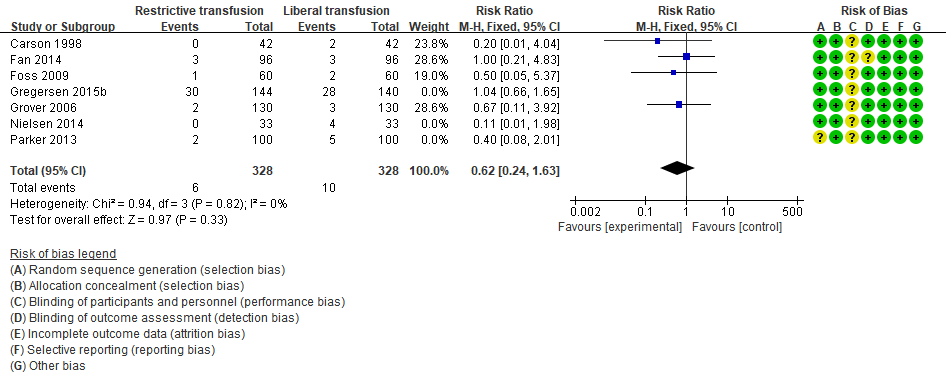


Supplementary figure 9 Forest plots depicting the comparison between the restrictive blood transfusion group (Threshold 7 to 8 g/dL) and liberal blood transfusion group (Threshold 9 to 10 g/dL): Lung infection.

**
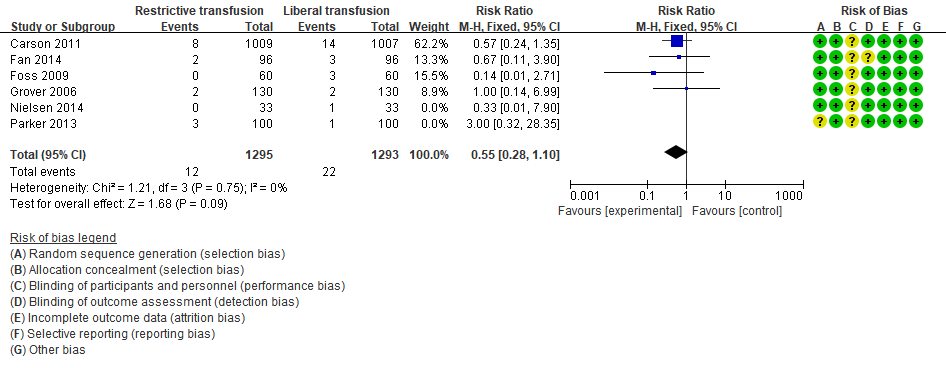
**

Supplementary figure 10 Forest plots depicting the comparison between the restrictive blood transfusion group (Threshold 7 to 8 g/dL) and liberal blood transfusion group (Threshold 9 to 10 g/dL): Wound infection.

**
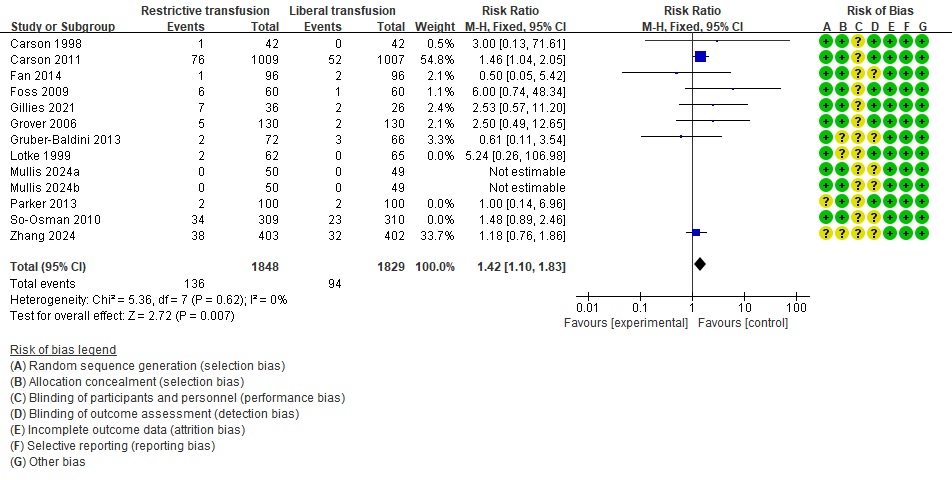
**

Supplementary figure 11 Forest plots depicting the comparison between the restrictive blood transfusion group (Threshold 7 to 8 g/dL) and liberal blood transfusion group (Threshold 9 to 10 g/dL): Cardiovascular events.

**
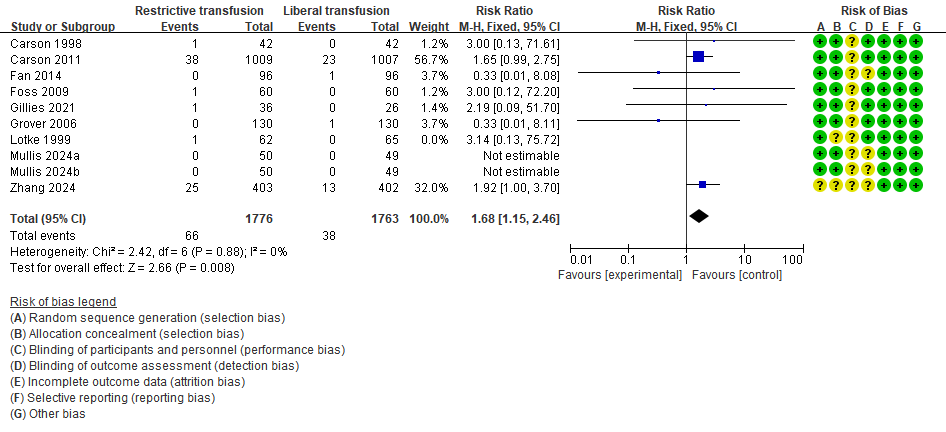
**

Supplementary figure 12 Forest plots depicting the comparison between the restrictive blood transfusion group (Threshold 7 to 8 g/dL) and liberal blood transfusion group (Threshold 9 to 10 g/dL): Myocardial infarction.

**
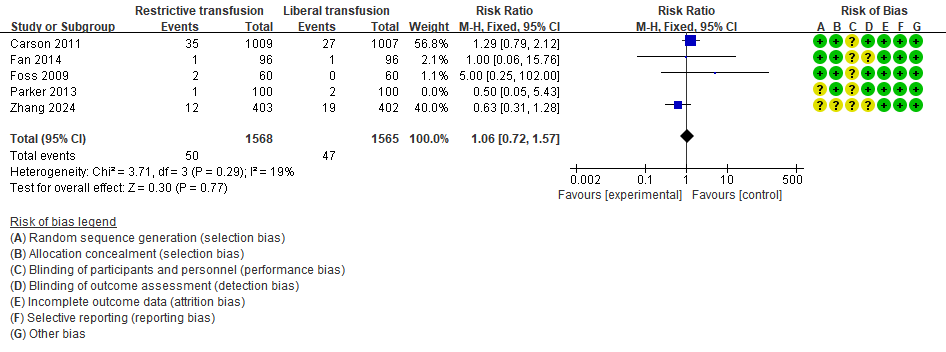
**

Supplementary figure 13 Forest plots depicting the comparison between the restrictive blood transfusion group (Threshold 7 to 8 g/dL) and liberal blood transfusion group (Threshold 9 to 10 g/dL): Congestive heart failure.

**
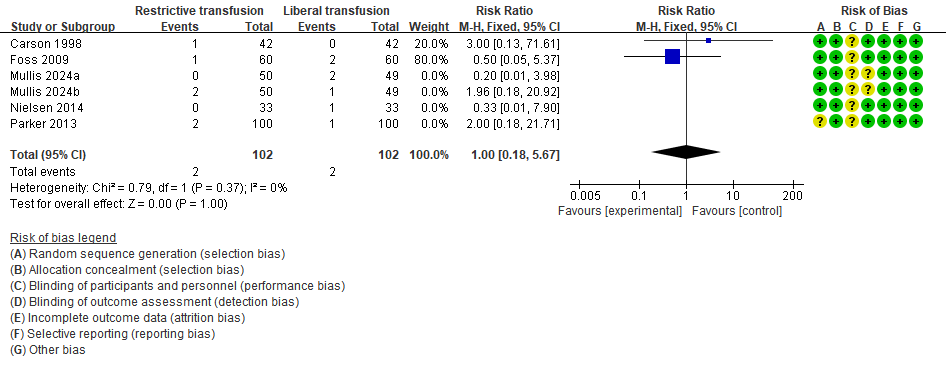
**

Supplementary figure 14 Forest plots depicting the comparison between the restrictive blood transfusion group (Threshold 7 to 8 g/dL) and liberal blood transfusion group (Threshold 9 to 10 g/dL): Thromboembolic events.

**
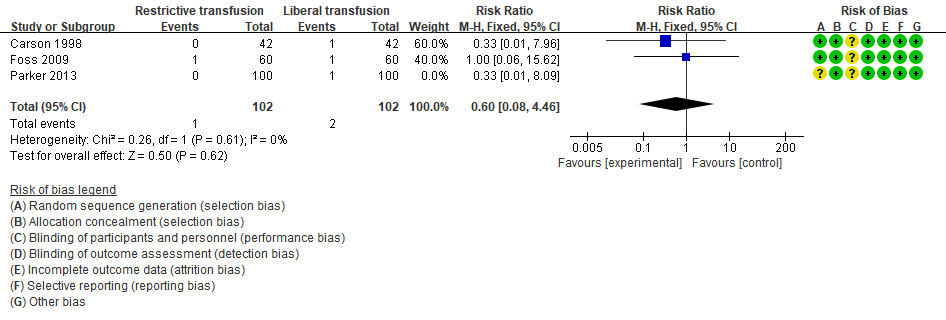
**

Supplementary figure 15 Forest plots depicting the comparison between the restrictive blood transfusion group (Threshold 7 to 8 g/dL) and liberal blood transfusion group (Threshold 9 to 10 g/dL): Cerebrovascular accidents.

**
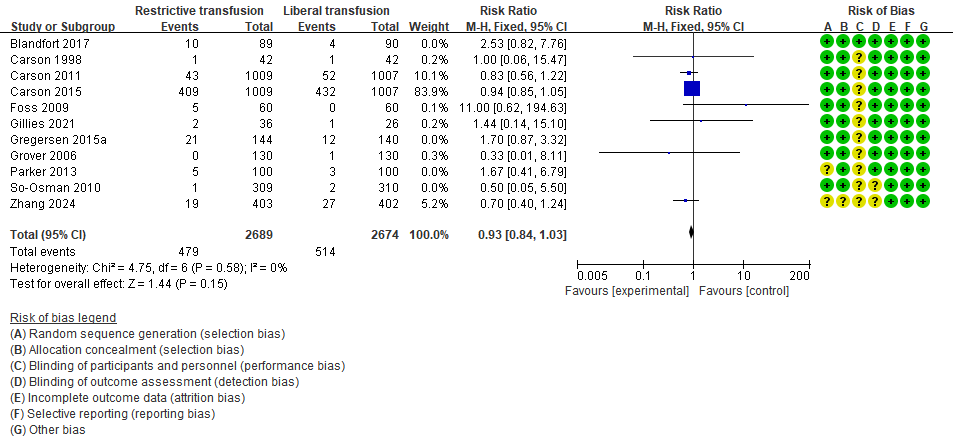
**

Supplementary figure 16 Forest plots depicting the comparison between the restrictive blood transfusion group (Threshold 7 to 8 g/dL) and liberal blood transfusion group (Threshold 9 to 10 g/dL): Mortality (≤30 days).

**
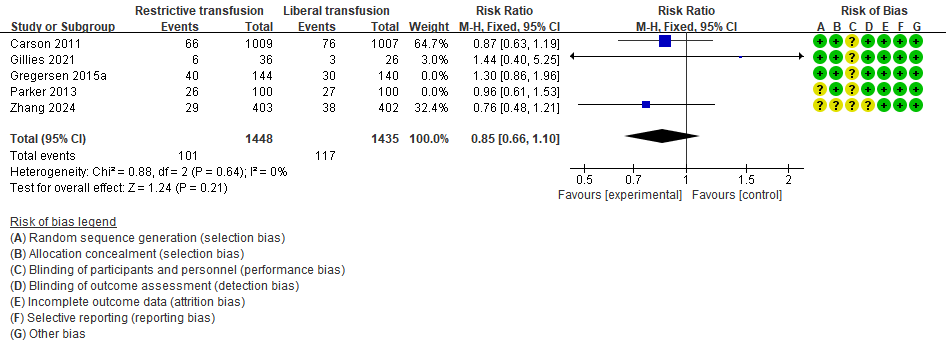
**

Supplementary figure 17 Forest plots depicting the comparison between the restrictive blood transfusion group (Threshold 7 to 8 g/dL) and liberal blood transfusion group (Threshold 9 to 10 g/dL): Mortality (≥60 days).

**
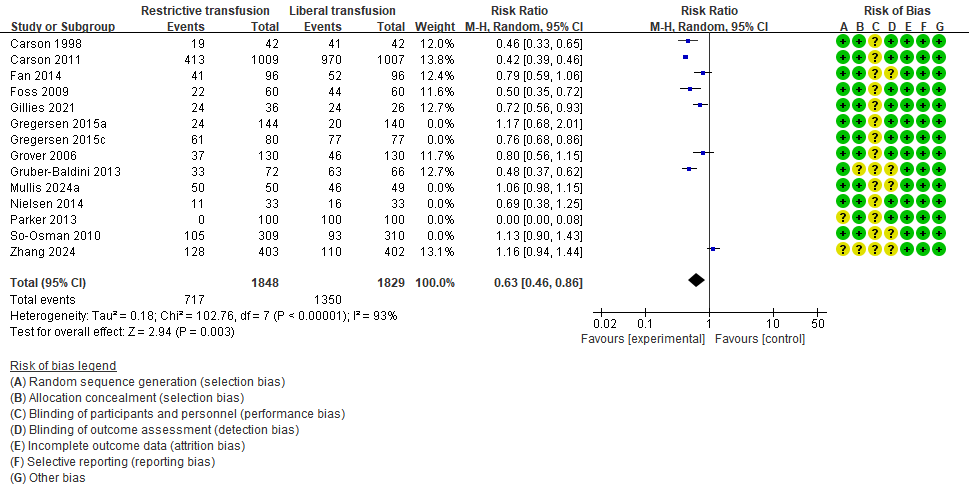
**

Supplementary figure 18 Forest plots depicting the comparison between the restrictive blood transfusion group (Threshold 7 to 8 g/dL) and liberal blood transfusion group (Threshold 9 to 10 g/dL): Transfusion rates.

**
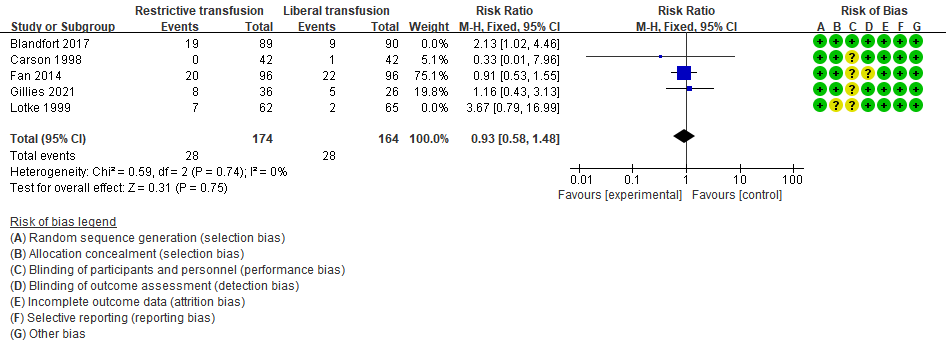
**

Supplementary figure 19 Forest plots depicting the comparison between the restrictive blood transfusion group (Threshold 7 to 8 g/dL) and liberal blood transfusion group (Threshold 9 to 10 g/dL): Delirium.

**
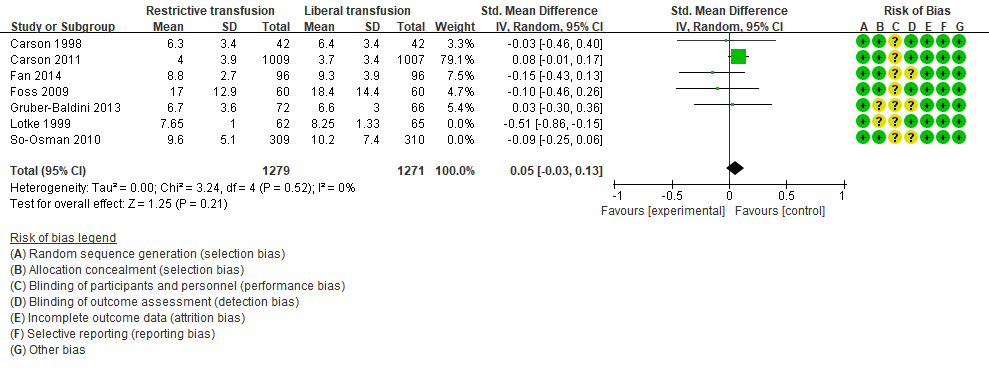
**

Supplementary figure 20 Forest plots depicting the comparison between the restrictive blood transfusion group (Threshold 7 to 8 g/dL) and liberal blood transfusion group (Threshold 9 to 10 g/dL): Length of hospitalization.


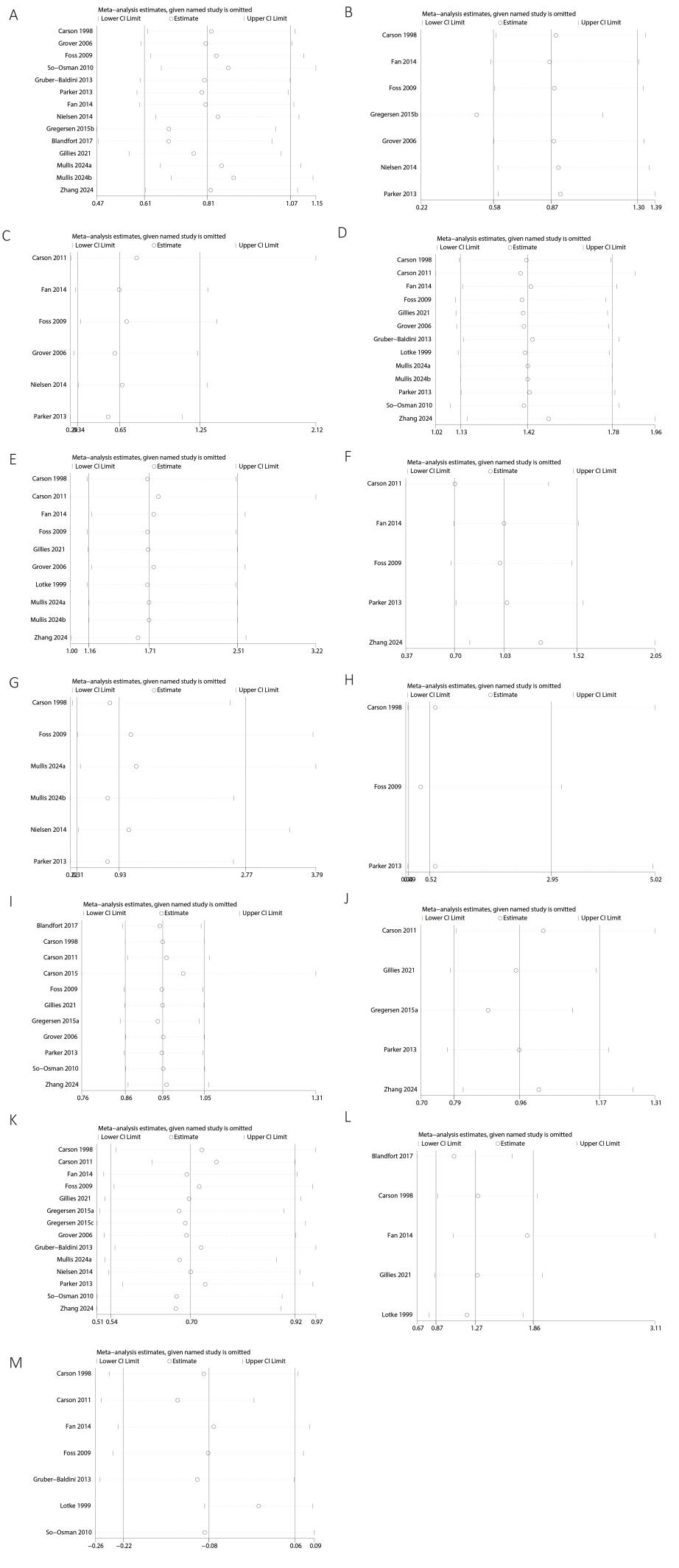


Supplementary figure 21 Sensitivity analysis results. (A) Overall infection; (B) Lung infection; (C) Wound infection; (D) Cardiovascular Events; (E) Myocardial infarction; (F) Congestive heart failure; (G) Thromboembolic Events; (H) Cerebrovascular Accidents; (I) Mortality (≤30 days); (J) Mortality (≥60 days); (K) Transfusion rates; (L) Delirium; (M) Length of hospitalization.


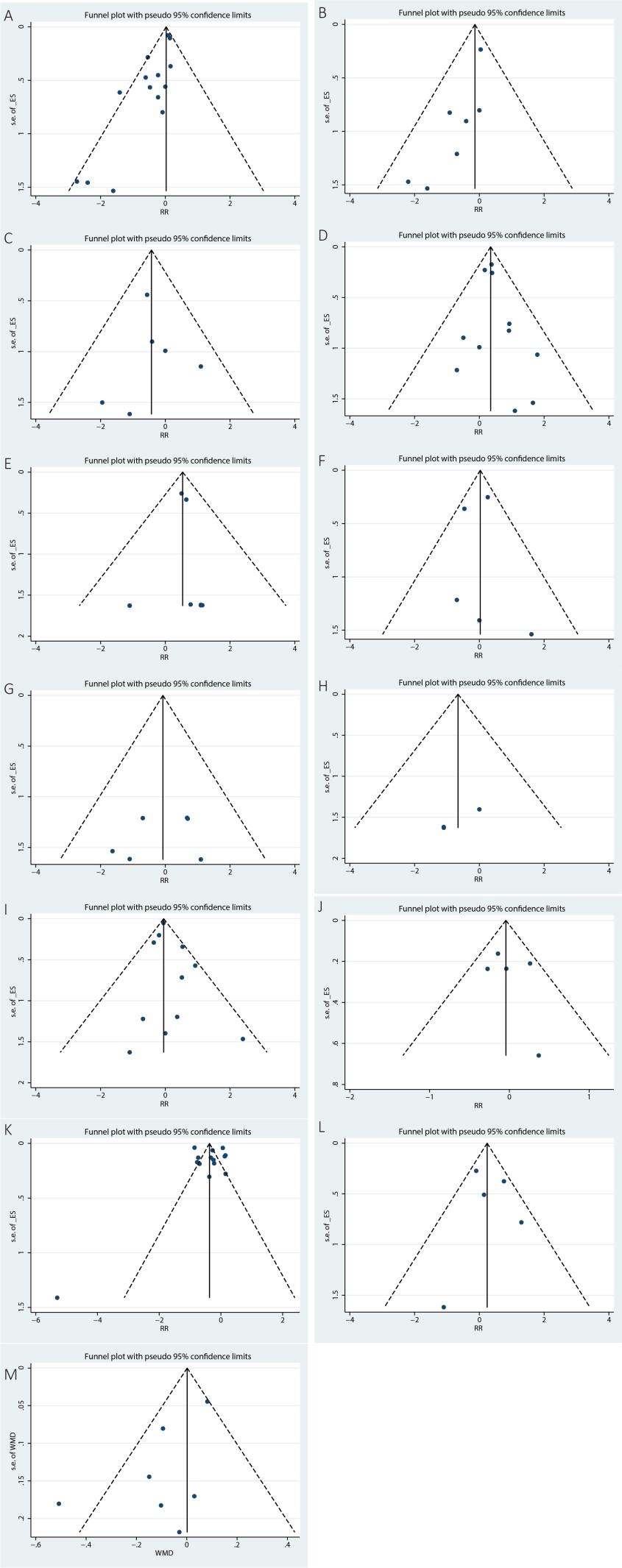


Supplementary figure 22 Funnel chart of publication bias. (A) Overall infection; (B) Lung infection; (C) Wound infection; (D) Cardiovascular Events; (E) Myocardial infarction; (F) Congestive heart failure; (G) Thromboembolic Events; (H) Cerebrovascular Accidents; (I) Mortality (≤30 days); (J) Mortality (≥60 days); (K) Transfusion rates; (L) Delirium; (M) Length of hospitalization.


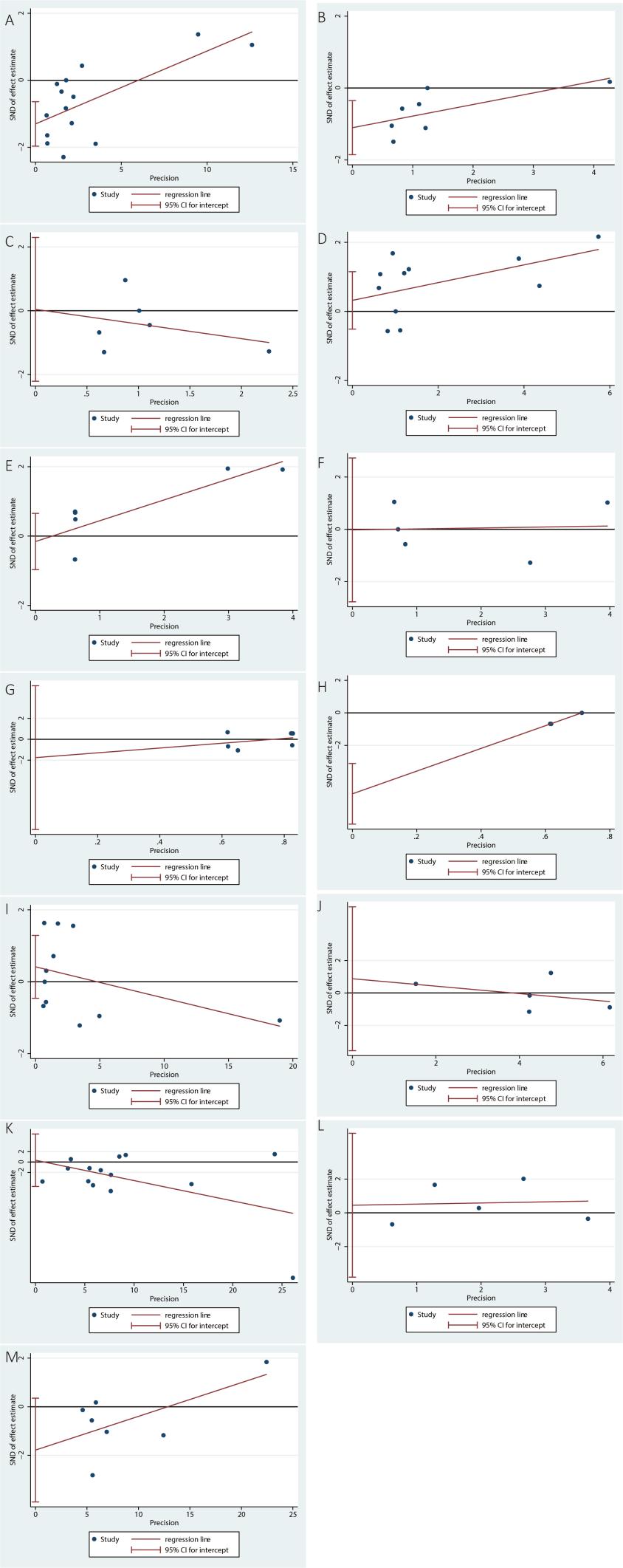


Supplementary figure 23 Results of egger’s test. (A) Overall infection; (B) Lung infection; (C) Wound infection; (D) Cardiovascular Events; (E) Myocardial infarction; (F) Congestive heart failure; (G) Thromboembolic Events; (H) Cerebrovascular Accidents; (I) Mortality (≤30 days); (J) Mortality (≥60 days); (K) Transfusion rates; (L) Delirium; (M) Length of hospitalization.


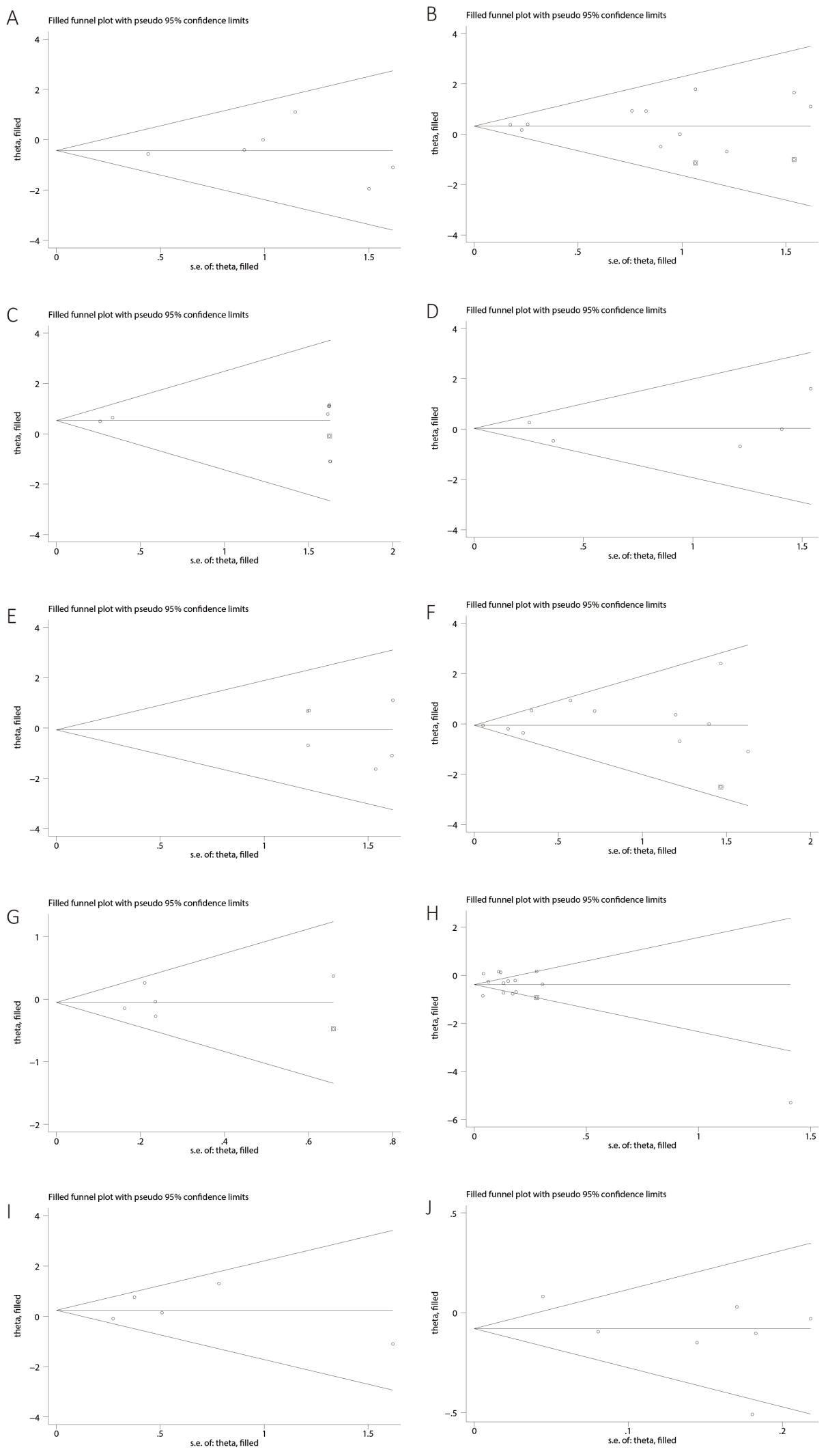


Supplementary figure 24 Trim and fill method results. (A) Wound infection; (B) Cardiovascular Events; (C) Myocardial infarction; (D) Congestive heart failure; (E) Thromboembolic Events; (F) Mortality (≤30 days); (G) Mortality (≥60 days); (H) Transfusion rates; (I) Delirium; (J) Length of hospitalization.


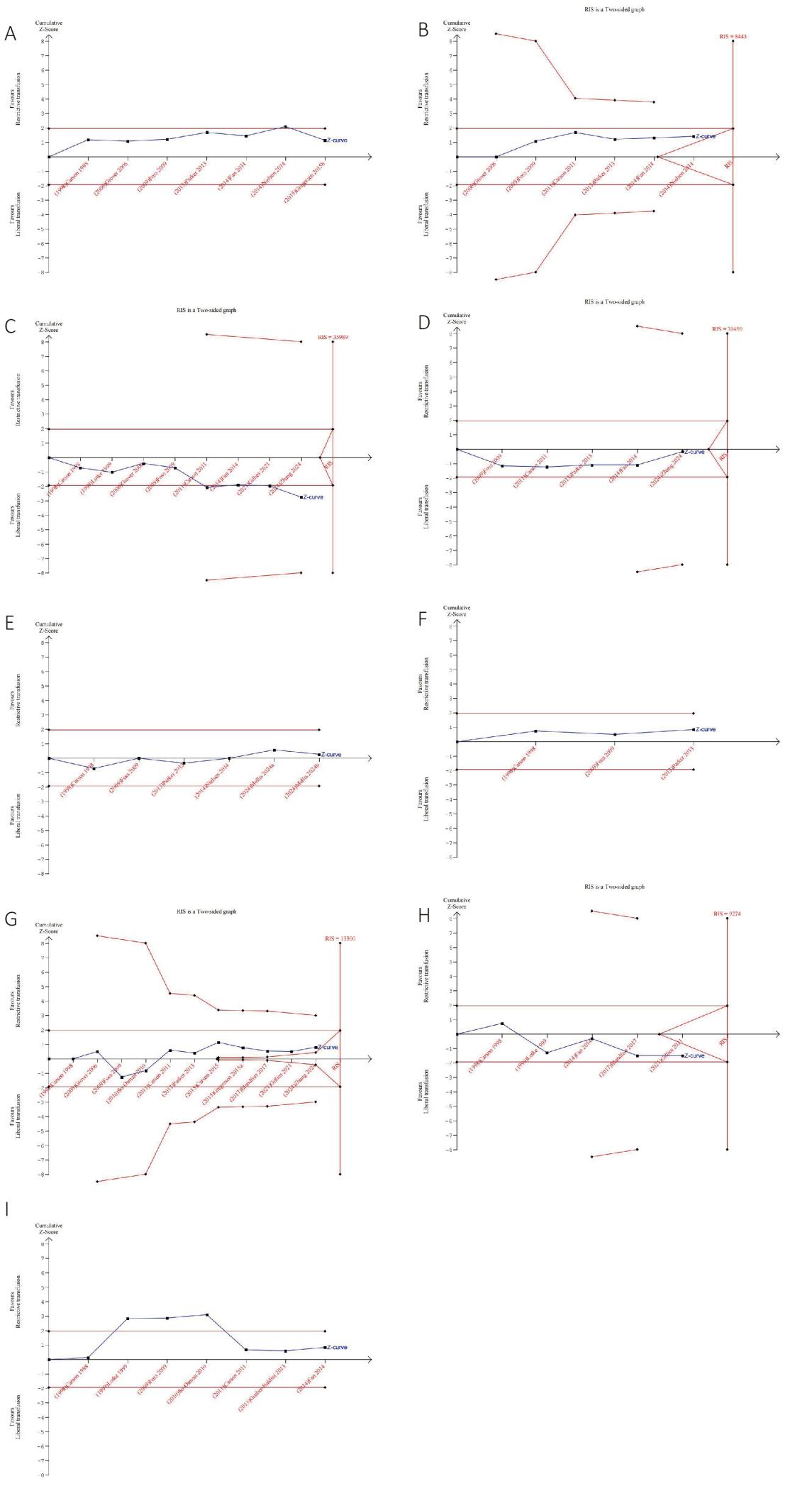


Supplementary figure 25 Trial sequential analysis. A diversity-adjusted information size was calculated based on using α = 0.05 (two-sided), β = 0.20 (power 80%), and an anticipated relative risk reduction (RRR) of 20%. (A) Lung infection; (B) Wound infection; (C) Myocardial infarction; (D) Congestive heart failure; (E) Thromboembolic Events; (F) Cerebrovascular Accidents; (G) Mortality (≤30 days); (H) Delirium; (I) Length of hospitalization.

**4. Supplementary Table**

Supplementary Table 1 Definition of cardiovascular disease in included studies.

| **Study** | **Outcome reported** | **Definition of cardiovascular disease** |
| --- | --- | --- |
| Carson 1998 | MI | Postoperative MI was defined as definite if the patient had positive CK-MB enzymes, if an electrocardiogram was interpreted locally as consistent with a MI, or if there was postmortem evidence of an acute MI. MI was defined as possible if the patient had chest pain and an electrocardiogram interpreted as consistent with possible new MI. |
| Lotke 1999 | MI, arrhythmia | NR |
| Grover 2006 | MI, left bundle branch block, ventricular tachycardia, cardiac arrest | Based on dynamic electrocardiogram, specific definition not reported. |
| Foss 2009 | MI, CHF, arrhythmia | NR |
| So-Osman 2010 | Cardiovascular events | NR |
| Carson 2011 | MI, CHF, angina | Detection of rise or fall of cardiac troponin I with at least one value above the 99th percentile of the upper reference limit in the context of myocardial ischemia and at least one of the following. Symptoms of myocardial ischemia; New ECG changes indicative of ischemia (e.g. ST-T changes or new left bundle branch block (LBBB)) or development of pathological Q waves. Imaging evidence of new loss of viable myocardium or new regional wall motion abnormality. |
| Zhang 2024 | MI，CHF, unstable angina | In-hospital myocardial infarction is characterized by any abnormal pattern of biomarkers, specifically cardiac troponin (I or T) levels that surpass 1.5 times the decision limit (the 99th percentile of a reference control group) at least once, accompanied by a rising or falling trend within the first 24 h following a suspected clinical event. Alternatively, it can be identified if CKMB (preferably CK-MB mass) exceeds 1.5 times the decision limit in two consecutive samples, along with ischemic symptoms, ECG changes suggesting ischemia, or imaging findings indicating new loss of viable myocardium or new regional wall motion abnormalities. Additionally, if cardiac biomarkers (troponin, CK-MB) are incomplete or unavailable, the emergence of pathological Q-waves on an ECG compared to baseline is also regarded as indicative of myocardial infarction |
| Gruber-Baldini 2013 | CHF | NR |
| Parker 2013 | CHF, Arrhythmia | NR |
| Fan 2014 | MI, CHF | NR |
| Gillies 2021 | MI | NR |

Abbreviation: MI, myocardial infarction; CHF, congestive heart failure; NR, not reported.

Supplementary Table 2 Definitions of patients at high risk for cardiovascular disease.

| **Study** | **Patient characteristics** | **Definition of patients at high risk for cardiovascular disease.** |
| --- | --- | --- |
| Carson 2011 | Hip fracture patients at high risk for cardiovascular disease | Patients with cardiovascular disease or any of the following cardiovascular risk factors: a history of or treatment for hypertension, diabetes mellitus, or hypercholesterolemia; a cholesterol level of 200 mg or more per deciliter or a low-density lipoprotein cholesterol level of 130 mg or more per deciliter; current tobacco use; or a creatinine level of more than 2.0 mg per deciliter. |
| Carson 2015 | Hip fracture patients at high risk for cardiovascular disease | Patients with cardiovascular disease or cardiovascular risk factors such as history of diabetes mellitus, hypertension, hypercholesterolemia, smoking, or creatinine concentration of 2 mg/dL or higher. |
| Zhang 2024 | Patients with coronary artery disease | Patients with coronary artery disease |
| Gruber-Baldini 2013 | Cardiovascular patients undergoing surgical hip fracture repair | Patients with cardiovascular disease or any of the following cardiovascular risk factors: a history of or treatment for hypertension, diabetes mellitus, or hypercholesterolemia; a cholesterol level of 200 mg or more per deciliter or a low-density lipoprotein cholesterol level of 130 mg or more per deciliter; current tobacco use; or a creatinine level of more than 2.0 mg per deciliter. |

In fact, in our subgroup analysis, we uniformly defined patients with cardiovascular disease and patients at risk for cardiovascular disease as patients at high risk for cardiovascular disease. We define the remaining study patients as ordinary patients. Although there may be some people at risk of cardiovascular disease, there are no special cardiovascular disease collective characteristics.

Supplementary Table 2 Process of the trim-and-fll method (flled meta-analysis)

| **Outcome** | **Method** | **Pooled Est** | **95% CI** | |  | **Asymptotic** | | | **Test for heterogeneity** | | | **Moment-based estimate of between studies variance** |
| --- | --- | --- | --- | --- | --- | --- | --- | --- | --- | --- | --- | --- |
|  |  |  | **Lower** | **Upper** |  | ***z* value** | ***P* value** | **No. of studies** | ***Q*** | ***degrees of freedom*** | ***p*** |  |
| Wound infection | Fixed | -0.427 | -1.081 | 0.227 |  | -1.280 | 0.200 | 6 | 3.248 | 5 | 0.662 | 0.000 |
|  | Random | -0.427 | -1.081 | 0.227 |  | -1.280 | 0.200 |  |  |  |  |  |
| Cardiovascular Events | Fixed | 0.325 | 0.100 | 0.549 |  | 2.832 | 0.005 | 13 | 8.925 | 12 | 0.709 | 0.000 |
|  | Random | 0.325 | 0.100 | 0.549 |  | 2.832 | 0.005 |  |  |  |  |  |
| Myocardial infarction | Fixed | 0.528 | 0.146 | 0.910 |  | 2.709 | 0.007 | 9 | 2.708 | 8 | 0.951 | 0.000 |
|  | Random | 0.528 | 0.146 | 0.910 |  | 2.709 | 0.007 |  |  |  |  |  |
| Congestive heart failure | Fixed | 0.029 | -0.363 | 0.422 |  | 0.147 | 0.883 | 5 | 4.069 | 4 | 0.397 | 0.005 |
|  | Random | 0.024 | -0.382 | 0.429 |  | 0.114 | 0.909 |  |  |  |  |  |
| Thromboembolic Event | Fixed | -0.073 | -1.163 | 1.017 |  | -0.131 | 0.896 | 6 | 2.993 | 5 | 0.701 | 0.000 |
|  | Random | -0.073 | -1.163 | 1.017 |  | -0.131 | 0.896 |  |  |  |  |  |
| Overall mortality ≤30days | Fixed | -0.052 | -0.149 | 0.044 |  | -1.064 | 0.287 | 12 | 14.423 | 11 | 0.210 | 0.035 |
|  | Random | -0.011 | -0.259 | 0.238 |  | -0.084 | 0.933 |  |  |  |  |  |
| Overall mortality ≥ 60 days | Fixed | -0.053 | -0.248 | 0.142 |  | -0.535 | 0.593 | 6 | 4.202 | 5 | 0.521 | 0.000 |
|  | Random | -0.053 | -0.248 | 0.142 |  | -0.535 | 0.593 |  |  |  |  |  |
| Transfusion rates | Fixed | -0.373 | -0.417 | -0.329 |  | -16.537 | 0.000 | 15 | 351.232 | 14 | 0.000 | 0.223 |
|  | Random | -0.386 | -0.647 | -0.124 |  | -2.889 | 0.004 |  |  |  |  |  |
| Delirium | Fixed | 0.241 | -0.141 | 0.623 |  | 1.237 | 0.216 | 5 | 5.964 | 4 | 0.202 | 0.116 |
|  | Random | 0.311 | -0.221 | 0.844 |  | 1.147 | 0.252 |  |  |  |  |  |
| Length of hospitalization | Fixed | 0.002 | -0.067 | 0.070 |  | 0.045 | 0.964 | 7 | 14.124 | 6 | 0.028 | 0.017 |
|  | Random | -0.079 | -0.218 | 0.060 |  | -1.115 | 0.265 |  |  |  |  |  |
